# Prognostic Implications of Left Ventricular Ejection Fraction Across the Flow‐Gradient Patterns in Severe Aortic Stenosis

**DOI:** 10.1161/JAHA.126.050412

**Published:** 2026-09-24

**Authors:** Tomohiko Taniguchi, Takeshi Morimoto, Yasuaki Takeji, Shinichi Shirai, Kenji Ando, Hiroyuki Tabata, Ko Yamamoto, Ryosuke Murai, Kohei Osakada, Kotaro Takahashi, Tomohisa Tada, Koichiro Murata, Yuki Obayashi, Yusuke Yoshikawa, Ryusuke Nishikawa, Masashi Amano, Takeshi Kitai, Chisato Izumi, Shojiro Tatsushima, Norio Kanamori, Makoto Miyake, Hiroyuki Nakayama, Masayasu Izuhara, Kazuya Nagao, Kenji Nakatsuma, Yutaka Furukawa, Moriaki Inoko, Masahiro Kimura, Mitsuru Ishii, Shunsuke Usami, Fumiko Nakazeki, Manabu Shirotani, Yasutaka Inuzuka, Koh Ono, Kenji Minatoya, Takeshi Kimura, the CURRENT AS Registry‐2 Investigators

**Affiliations:** ^1^ Kobe City Medical Center General Hospital Kobe Japan; ^2^ Hyogo Medical University Nishinomiya Japan; ^3^ Kanazawa University Graduate School of Medical Science Kanazawa Japan; ^4^ Kokura Memorial Hospital Kitakyushu Japan; ^5^ Kurashiki Central Hospital Kurashiki Japan; ^6^ Shizuoka General Hospital Shizuoka Japan; ^7^ Osaka Red Cross Hospital Osaka Japan; ^8^ Shizuoka City Shizuoka Hospital Shizuoka Japan; ^9^ Department of Cardiovascular Medicine Kyoto University Hospital Kyoto Japan; ^10^ National Cerebral and Cardiovascular Center Osaka Japan; ^11^ Japan Red Cross Wakayama Medical Center Wakayama Japan; ^12^ Shimada General Medical Center Shimada Japan; ^13^ Tenri Hospital Tenri Japan; ^14^ Hyogo Prefectural Amagasaki General Medical Center Amagasaki Japan; ^15^ Kishiwada City Hospital Kishiwada Japan; ^16^ Mitsubishi Kyoto Hospital Kyoto Japan; ^17^ Kitano Hospital Osaka Japan; ^18^ Koto Memorial Hospital Higashiomi Japan; ^19^ National Hospital Organization Kyoto Medical Center Kyoto Japan; ^20^ Kansai Electric Power Hospital Otsu Japan; ^21^ Japanese Red Cross Otsu Hospital Otsu Japan; ^22^ Kindai University Nara Hospital Ikoma Japan; ^23^ Shiga General Hospital Moriyama Japan; ^24^ Department of Cardiovascular Surgery Kyoto University Hospital Kyoto Japan; ^25^ Hirakata Kohsai Hospital Hirakata Japan

**Keywords:** aortic stenosis, high‐gradient aortic stenosis, left ventricular ejection fraction, low flow, low‐gradient aortic stenosis, normal flow, low‐gradient aortic stenosis, Valvular Heart Disease, Aortic Valve Replacement/Transcather Aortic Valve Implantation

## Abstract

**Background:**

Current guidelines classify normal‐flow low‐gradient (LG) aortic stenosis (AS) with left ventricular ejection fraction (LVEF) ≥50% as “moderate.” However, the prognostic impact of LVEF remains incompletely understood across various hemodynamic subtypes of AS.

**Methods:**

We analyzed 3363 consecutive patients with severe AS from the CURRENT AS Registry‐2 (Contemporary Outcomes After Surgery and Medical Treatment in Patients With Severe Aortic Stenosis). Patients were classified into LG AS with normal flow (N=1005), and LG AS with low flow (N=372), and high‐gradient AS (N=1986). Patients were further divided according to baseline LVEF: <50%, 50% to 59%, and ≥60%. The primary outcome measure was a composite of death or heart failure hospitalization. Within each hemodynamic subtype, clinical outcomes were compared across baseline LVEF categories of <50%, 50% to 59%, and ≥60% (reference), and unadjusted and adjusted hazard ratios (aHRs) were estimated using Cox proportional hazards models. The median follow‐up period in the entire cohort was 763 days.

**Results:**

LVEF <50% was associated with a greater hazard of death or heart failure hospitalization than LVEF ≥60% in all 3 subtypes of AS. LVEF 50% to 59% was associated with a greater hazard of death or heart failure hospitalization than LVEF ≥60% in LG AS with normal flow (aHR, 1.80 [95% CI, 1.35–2.40], *P*<0.001) and high‐gradient AS (aHR, 1.42 [95% CI, 1.13–1.79], *P*=0.003) with hazards comparable to those with LVEF <50% but not in LG AS with low flow (aHR, 1.13 [95% CI, 0.72–1.79], *P*=0.59).

**Conclusions:**

These findings suggest that LVEF may provide additional prognostic stratification within severe AS phenotypes, particularly in normal‐flow LG AS.

**Registration:**

URL: https://center6.umin.ac.jp; Unique Identifier: UMIN000034169.

## Graphical abstract

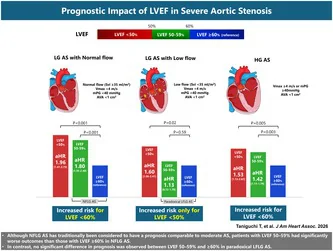

**Nonstandard Abbreviations and Acronyms**
AS: aortic stenosis
AVA: aortic valve area
HG: high gradient
LG: low gradient
NF: normal flow
PG: pressure gradient

**Clinical Perspective**

**What Is New?**

- In a nationwide contemporary registry of 3363 patients with severe aortic stenosis, left ventricular ejection fraction (LVEF) 50% to 59% (versus ≥60%) was independently associated with a higher hazard of death or heart failure hospitalization in low‐gradient aortic stenosis with normal flow and in high‐gradient aortic stenosis.
- In contrast, we did not detect a significant prognostic separation between patients with LVEF 50% to 59% and those with LVEF ≥60% in low‐gradient aortic stenosis with low flow, whereas LVEF <50% remained associated with significantly worse prognosis.

**What Are the Clinical Implications?**

- Incorporating an LVEF threshold of 60% into risk stratification may refine the prognostic classification of severe AS beyond gradient and flow criteria and help identify higher‐risk patients within the normal‐flow low‐gradient phenotype.

The assessment of disease severity in aortic stenosis (AS) can be particularly challenging in patients with low‐gradient (LG) AS, which complicates both diagnosis and therapeutic decision making. In the 2025 European Society of Cardiology/European Association for Cardio‐Thoracic Surgery guidelines,^1^ among LG AS, the subset with preserved left ventricular ejection fraction (LVEF) and normal flow (NF), which is referred to as NFLG AS, is regarded as “unlikely to be severe AS” even though it met the hemodynamic criteria for severe AS with an aortic valve area (AVA) <1 cm^2^. In contrast, the 2020 American College of Cardiology/American Heart Association guidelines did not provide a clear statement regarding this entity.^2^ The cutoff value of LVEF for LV systolic dysfunction in patients with severe AS has traditionally been set at 50%.^1^, ^2^ However, this cutoff has been based on limited evidence and lacks a firm scientific foundation. More recently, several studies have suggested that a higher threshold may be more appropriate.^3^, ^4^, ^5^, ^6^ However, no previous studies have evaluated the prognostic impact of LVEF 50% to 59% in LG AS with NF or other hemodynamic subtypes of severe AS.

Therefore, we compared clinical and echocardiographic characteristics as well as clinical outcomes across LVEF categories (<50%, 50%–59%, and ≥60%) within each hemodynamic subtype (LG AS with NF, LG AS with low‐flow and high‐gradient [HG] AS) in a large Japanese database of consecutive patients with severe AS.

## METHODS

Based on the confidentiality agreement related to the data, we are unable to make the analytical data accessible to readers.

### Study Population

The CURRENT AS (Contemporary Outcomes After Surgery and Medical Treatment in Patients With Severe Aortic Stenosis) Registry‐2 is a prospective multicenter registry conducted among 21 participating centers in Japan (Supplemental Material: Appendix). The registry design and enrollment procedures have been described previously.^7^ Briefly, 3369 consecutive patients were enrolled between April 2018 and December 2020 when they were first identified as having severe AS, defined by at least 1 of the following echocardiographic criteria: peak aortic jet velocity (Vmax) >4.0 m/s, mean aortic pressure gradient (PG) >40 mm Hg, or AVA <1.0 cm^2^. We excluded patients with a history of percutaneous aortic balloon valvuloplasty or AV surgery. The study protocol was approved by the institutional review board of each participating center. Written informed consent was obtained from each patient at 19 centers, and at the remaining 2 centers, an opt‐out strategy was permitted by the respective institutional review boards. Management after the index echocardiographic examination was determined in routine clinical practice through discussion between the treating physician and patient, with heart‐team consultation when appropriate. Patients were managed either with an initial AV replacement (AVR) strategy or conservatively.

For the present study, 6 of the 3369 registered patients were excluded: 1 because baseline LVEF was unavailable and 5 because stroke volume index was unavailable in the setting of LG AS. The resulting 3363 patients were categorized into the 3 hemodynamic strata according to baseline flow and gradient: LG AS with NF, defined as Vmax <4 m/s, mean aortic PG <40 mm Hg, AVA <1 cm^2^, and indexed stroke volume ≥35 mL/m^2^ (n=1005); LG AS with low flow, defined as Vmax <4 m/s, mean aortic PG <40 mm Hg, AVA <1 cm^2^, and indexed stroke volume <35 mL/m^2^ (n=372); HG AS, defined as Vmax ≥4 m/s or mean PG ≥40 mm Hg (n=1986) (Figure 1). In the current guidelines, the term “NFLG AS” refers to LG AS with NF and LVEF ≥50%.^1^ In contrast, in the present study, “LG AS with NF” was defined solely by gradient and flow criteria irrespective of LVEF. To avoid confusion, we used “LG AS with NF” for the hemodynamic category overall, and “NFLG AS” only when referring to the guideline‐defined subgroup with LVEF ≥50%. The study population was further subdivided into 3 groups according to LVEF at the index echocardiography in each subtype of AS; LG AS with NF (<50% in 133 patients, 50%–59% in 201 patients, and ≥60% in 671 patients); LG AS with low flow (<50% in 152 patients, 50%–59% in 72 patients, and ≥60% in 148 patients); HG AS (<50% in 207 patients, 50%–59% in 341 patients, and ≥60% in 1438 patients). Follow‐up commenced on the day of the index echocardiography. The follow‐up data for this analysis were collected at 1 year after enrollment of the last patient.

**Figure 1:**
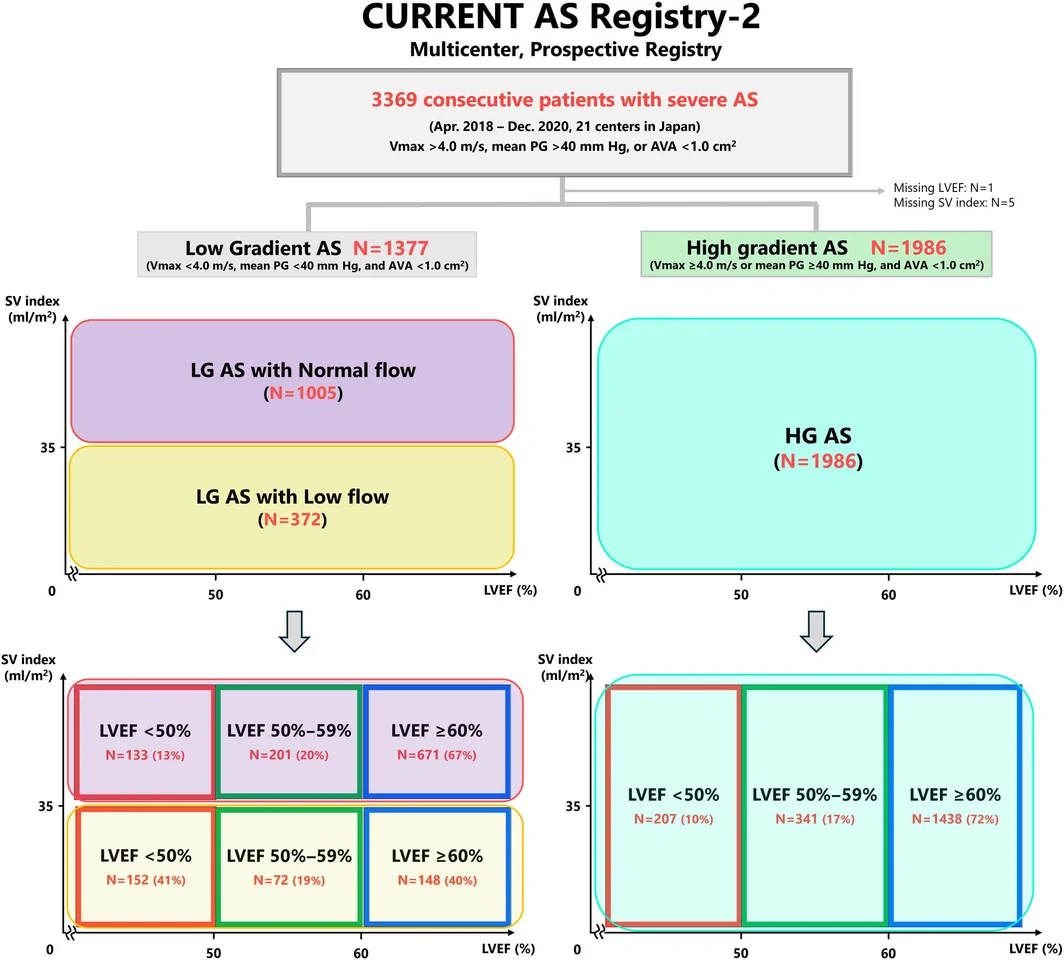
Study flow chart. AS indicates aortic stenosis; AVA, aortic valve area; CURRENT AS, Contemporary Outcomes After Surgery and Medical Treatment in Patients With Severe Aortic Stenosis; HG, high gradient; LG, low gradient; LVEF, left ventricular ejection fraction; PG, pressure gradient; SV, stroke volume; and Vmax, peak aortic jet velocity.

### Echocardiographic and Computed Tomographic Evaluation

Transthoracic echocardiography was performed locally at each participating institution using 2‐dimensional and Doppler techniques. To reduce interinstitutional variability in the assessment of AS severity, registry‐specific workshops and hands‐on training sessions were provided for participating echocardiographic technicians. In each participating center, we highly recommended the use of multiple acoustic windows (apical, high parasternal, or right parasternal) to obtain the greatest peak velocity in accordance with the guidelines. We evaluated the extent of cardiac damage on echocardiography according to the previously proposed staging scheme in Supplemental Methods: Data S1.^8^ We also recommended computed tomography (CT) assessment of AV calcification particularly in patients with LG AS. The CT images were analyzed using 3mensio Structural Heart 10.4 (Pie Medical Imaging). Details of echocardiographic and CT examination were described in Supplemental Methods: Data S1.

### Outcome Measures

The primary outcome measure for this analysis was a composite of all‐cause death or heart failure (HF) hospitalization. HF hospitalization was defined as hospitalization due to worsening HF treated with intravenous drug therapy. Other clinical event definitions are provided in the Supplemental Methods: Data S2. All reported clinical events underwent adjudication by the registry clinical event committee.

### Statistical Analysis

Baseline clinical, echocardiographic, and CT variables were examined across the 3 LVEF categories separately within each group with hemodynamic AS. Categorical data were reported as numbers and percentages, and compared using the chi‐square or the Fisher's exact test, as appropriate. Continuous data were reported as the mean±SD or median and interquartile range (IQR) according to their distribution and were compared using the Student *t* test or Wilcoxon rank‐sum test. Cumulative incidence was estimated by the Kaplan–Meier method and the differences among LVEF <50%, LVEF 50% to 59%, and LVEF ≥60% (reference) were assessed by the log‐rank test within each of the 3 hemodynamic subtypes of AS. Because the primary objective was to characterize the prognostic implications of LVEF within individual hemodynamic phenotypes, the primary analyses did not include interaction testing between LVEF category and AS subtype. In the multivariable Cox proportional hazard models, we used 22 clinically relevant covariates listed in Table1 including the initial treatment strategy as the risk‐adjusting variables and the centers were incorporated as the stratification variable, consistent with our previous report.^9^ The proportional hazards assumption was examined for each risk‐adjusting variable using plots of log (time) versus log [−log (survival)] stratified by the variable, with no meaningful violation identified. Among the risk‐adjusting variables, missing data were restricted to body mass index, which was unavailable in 11 patients. Because we considered the effect of missing data was minor, we constructed the multivariable models with those without missing data.

**Table 1:** Baseline Clinical and Echocardiographic Characteristics According to the Baseline Left Ventricular Ejection Fraction: LG AS with Normal Flow

LG AS with normal flow	LVEF <50%	LVEF 50%–59%	LVEF ≥60% (reference)	LVEF <50% vs LVEF ≥60%	LVEF 50%–59% vs LVEF ≥60%	Missing value
	No.=133	No.=201	No.=671			
Clinical characteristics						
Age,* y	82.3±7.8	81.9±9.1	82.2±7.8	0.86	0.62	0
Men*	66 (50%)	88 (44%)	204 (30%)	<0.001	<0.001	0
Body mass index,* kg/m^2^	21.5±3.1	22.2±3.6	22.5±3.7	0.004	0.40	4
Hypertension*	109 (82%)	162 (81%)	569 (85%)	0.41	0.16	0
Current smoking*	5 (4%)	7 (3%)	27 (4%)	0.89	0.73	0
Diabetes	63 (47%)	72 (36%)	189 (28%)	<0.001	0.04	0
On insulin therapy*	13 (10%)	12 (6%)	38 (6%)	0.08	0.87	0
Prior myocardial infarction*	42 (32%)	32 (16%)	44 (7%)	<0.001	<0.001	0
Coronary artery disease*	88 (66%)	94 (47%)	220 (33%)	<0.001	<0.001	0
Prior symptomatic stroke*	31 (23%)	33 (16%)	102 (15%)	0.022	0.68	0
Atrial fibrillation or flutter*	37 (28%)	55 (27%)	117 (17%)	0.005	0.002	0
Aortic/peripheral vascular disease*	15 (11%)	23 (11%)	61 (9%)	0.43	0.32	0
Creatinine level >2 mg/dL and nondialysis*	10 (8%)	7 (3%)	21 (3%)	0.02	0.80	0
Dialysis*	29 (22%)	25 (12%)	52 (8%)	<0.001	0.04	0
Anemia* ^,^ †	83 (62%)	116 (58%)	313 (47%)	<0.001	0.006	0
Liver cirrhosis (Child–Pugh class B/C)	0 (0%)	2 (1.0%)	4 (0.6%)	0.37	0.55	0
Malignancy currently under treatment*	5 (4%)	14 (7%)	30 (4%)	0.71	0.16	0
Chronic lung disease (moderate or severe)*	6 (5%)	18 (9%)	29 (4%)	0.92	0.01	0
Society of Thoracic Surgeons score (predicted risk of mortality), %	6.0 (4.2–8.6)	4.9 (2.9–7.8)	4.2 (2.8–5.9)	<0.001	0.005	0
Symptoms possibly related to AS	86 (65%)	87 (43%)	239 (36%)	<0.001	0.049	0
Heart failure hospitalization at the index echocardiography*	27 (20%)	10 (5%)	22 (3%)	<0.001	0.26	0
Clinical frailty scale* ^,^ ‡						
1–3	56 (42%)	88 (44%)	336 (50%)	0.14	0.24	0
4–6	63 (47%)	100 (50%)	289 (43%)			
7–9	14 (11%)	13 (6%)	46 (7%)			
BNP, pg/mL	N=92	N=132	N=415			366
	365 (175–783)	162 (71–385)	85 (46–192)	<0.001	<0.001	
N‐terminal proBNP, pg/mL	N=15	N=25	N=74			891
	3905 (2018–19 567)	632 (345–2948)	425 (179–1014)	<0.001	0.049	
Cause of aortic stenosis						
Degenerative	125 (94%)	190 (95%)	640 (95%)	0.36	0.65	0
Congenital (unicuspid, bicuspid, or quadricuspid)	6 (5%)	9 (4%)	28 (4%)			
Rheumatic	2 (2%)	2 (1%)	3 (0.5%)			
Echocardiographic variables						
Peak aortic jet velocity, m/s	3.31±0.39	3.45±0.36	3.54±0.32	<0.001	<0.001	0
Mean aortic pressure gradient, mm Hg	25.8±6.4	27.5±6.2	28.5±6.0	<0.001	0.045	2
AVA (equation of continuity), cm^2^	0.82±0.12	0.83±0.11	0.85±0.10	0.004	0.02	0
Indexed AVA, cm^2^/m^2^	0.56±0.09	0.56±0.08	0.58±0.09	0.002	0.003	4
LV end‐diastolic diameter, mm	50.8±6.8	45.1±5.7	42.6±5.1	<0.001	<0.001	0
LV end‐systolic diameter, mm	39.6±7.2	30.8±4.9	26.5±3.9	<0.001	<0.001	3
LVEF, %	40±7	56±3	67±5	<0.001	<0.001	0
LV remodeling pattern						7
Concentric hypertrophy	23/132 (17%)	64/201 (32%)	162/665 (24%)	<0.001	<0.001	
Concentric remodeling	10/132 (8%)	47/201 (23%)	251/665 (38%)			
Eccentric hypertrophy	77/132 (58%)	36/201 (18%)	75/665 (11%)			
Normal	22/132 (17%)	54/201 (27%)	177/665 (27%)			
LV outflow tract, mm	21.0±1.9	20.5±1.9	19.9±1.7	<0.001	<0.001	0
Stroke volume index, mL/m^2^	41±5	44±6	47±8	<0.001	<0.001	4
Septal E/e′ >15	84/102 (82%)	111/166 (67%)	360/578 (62%)	<0.001	0.28	159
Cardiac damage						
Stage 0	0	19 (9%)	112 (17%)	<0.001	0.01	0
Stage 1	28 (21%)	35 (17%)	148 (22%)			
Stage 2	85 (64%)	124 (62%)	342 (51%)			
Stage 3–4	20 (15%)	23 (11%)	69 (10%)			
Any combined valvular disease						
Moderate or severe*	53 (40%)	51 (25%)	140 (21%)	<0.001	0.18	0
Moderate or severe aortic regurgitation	20 (15%)	20 (10%)	45 (7%)	0.001	0.12	0
Moderate or severe mitral stenosis	2 (2%)	5 (2%)	14 (2%)	0.66	0.73	0
Moderate or severe mitral regurgitation	33 (25%)	24 (12%)	50 (7%)	<0.001	0.045	0
Moderate or severe TR	20 (15%)	20 (10%)	68 (10%)	0.10	0.94	0
TR pressure gradient ≥40 mm Hg*	14 (11%)	19 (9%)	41 (6%)	0.07	0.10	0
Computed tomography variables	N=35	N=48	N=171			
Agatston score of aortic valve >2000 AU in men, and >1200 AU in women	12/35 (34%)	18/48 (38%)	74/171 (43%)	0.33	0.47	751
Treatment strategy						
Initial SAVR or TAVR strategy*	43 (32%)	55 (27%)	140 (21%)	0.004	0.052	0
Initial SAVR	13 (10%)	20 (10%)	47 (7%)		…	
Initial TAVR	30 (23%)	35 (17%)	93 (14%)		…	
Conservative strategy	90 (68%)	146 (73%)	531 (79%)	0.004	0.052	0
Watchful waiting	58 (44%)	109 (54%)	440 (66%)		…	
Patient rejection	12 (9%)	13 (6%)	44 (7%)		…	
Contraindication for both SAVR and TAVR	20 (15%)	24 (12%)	47 (7%)	…	…	

Categorical variables were presented as numbers and percentages. Continuous variables were expressed as the mean±SD or median and interquartile range. The definitions of LV geometry, and cardiac damage stages were listed in the Supplementary File. AS indicates aortic stenosis; AU, arbitrary units; AVA, aortic valve area;BNP, brain natriuretic peptide; HF, heart failure; LG, low gradient; LV, left ventricular; LVEF, left ventricular ejection fraction; SAVR, surgical aortic valve replacement; TAVR, transcatheter aortic valve replacement; and TR, tricuspid regurgitation.

* Risk‐adjusting variables selected for the Cox proportional hazard model.

^†^ Anemia was defined by the World Health Organization criteria (hemoglobin <12.0 g/dL in women and <13.0 g/dL in men).

^‡^ Clinical frailty scale scores were determined according to the Canadian Study of Health and Aging grading criteria.

Unadjusted and adjusted hazard ratios (HR) and their 95% CI were estimated using Cox proportional hazard models in the entire study population and within the initial AVR and conservative strata. In each model, LVEF category was represented by 2 indicator variables for LVEF <50% and LVEF 50% to 59%, with LVEF ≥60% as the reference category. As an exploratory analysis, we evaluated the effects of LVEF on clinical outcomes in patients without prior myocardial infarction and in patients whose LVEF was measured using the biplane Simpson method of disks. All reported *P* values were 2 tailed, and *P* values <0.05 were considered statistically significant. Considering the exploratory nature of this study, *P* values were not adjusted for multiple testing, and *P* values should be interpreted as exploratory rather than as confirmatory tests of statistical significance. Statistical analyses were conducted by 2 physicians (Tomohiko Taniguchi and Yasuaki Takeji) and a statistician (Takeshi Morimoto) with the use of JMP 17, R version 3.6.1, or SAS 9.4.

## RESULTS

### Baseline Clinical Characteristics According to LVEF in LG AS With Normal Flow

Among 1005 patients with LG AS with NF, baseline characteristics were significantly different across the LVEF groups (Table1). Compared with patients with LVEF ≥60%, those with LVEF <50% or 50% to 59% were more often men, and more frequently had cardiovascular comorbidities such as prior myocardial infarction, coronary artery disease, atrial fibrillation, and dialysis. At index echocardiography, patients with LVEF <50% compared with those with LVEF ≥60% were more frequently symptomatic and more often presented with acute HF hospitalization, whereas these findings were not observed in the group with LVEF 50% to 59%. Estimated surgical risks scores were higher in both lower LVEF groups compared with the LVEF ≥60% group. Regarding echocardiographic parameters, both lower LVEF groups had smaller Vmax, AVA, and stroke volume index, and larger LV size and LV outflow tract diameter compared with the LVEF ≥60% group. Stroke volume index showed a gradual decline from the group with LVEF ≥60% to the group with LVEF 50% to 59% and <50%. The AV calcification score by CT was not significantly different across the LVEF groups (Table1).

### Clinical Outcomes According to LVEF in LG AS With Normal Flow

The median follow‐up period in the entire cohort was 763 (interquartile range, 426–1059) days. The cumulative 3‐year incidence of surgical AV replacement or transcatheter AV replacement did not differ significantly across LVEF categories (Figure S1). The cumulative 3‐year incidence of the primary outcome was significantly higher in the low LVEF groups (<50% and 50%–59%) than in the LVEF ≥60% group (56.4%, 48.9%, and 31.4%, respectively) (Figures 2A and 3A). After adjusting for confounders including the initial treatment strategies, both LVEF <50% and LVEF 50% to 59% were associated with greater hazards of the primary outcome compared with LVEF ≥60% (HR, 1.96 [95% CI, 1.41–2.73], *P* < 0.001; HR, 1.80 [95% CI, 1.35–2.40], *P* < 0.001, respectively) with similar magnitudes of association between the 2 lower LVEF groups. Similar associations were observed for the individual components of the primary outcome (Figure 3A). The greater hazard associated with LVEF <50% and 50% to 59% relative to LVEF ≥60% for the primary outcome in the conservative stratum was consistent with the main analysis, whereas the negative effect of lower LVEF was markedly attenuated in the initial AVR stratum in patients with LG AS with NF (Figure S2).

**Figure 2:**
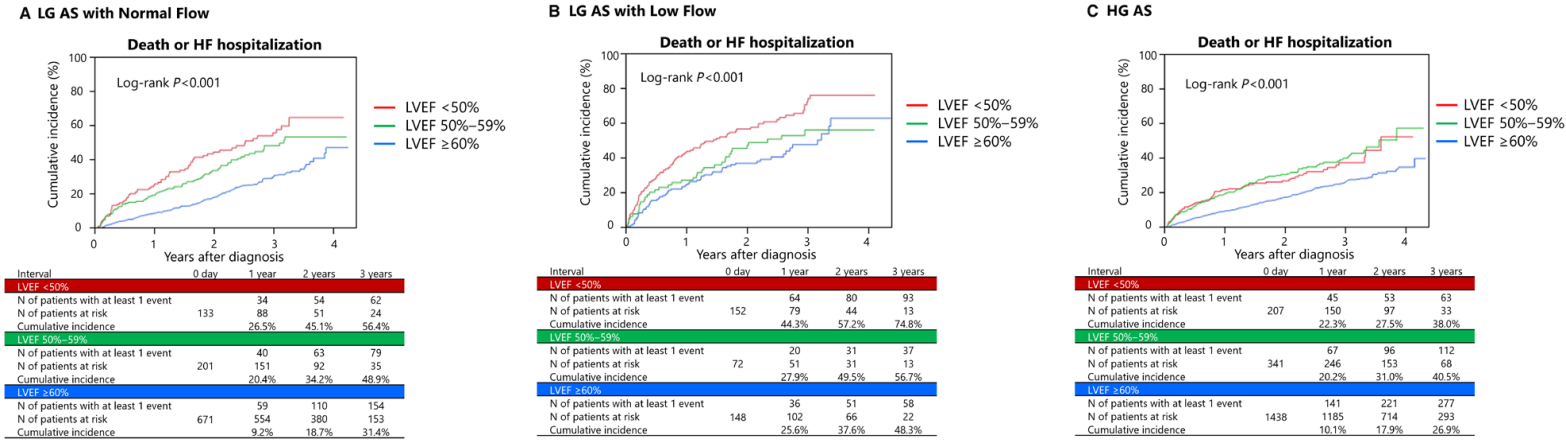
Kaplan–Meier curves for a composite of death or heart failure hospitalization according to the baseline left ventricular ejection fraction. **A**, LG AS with normal flow, **B**, LG with low flow, and **C**, HG AS. AS indicates aortic stenosis; HF, heart failure; HG, high gradient; LG, low gradient; and LVEF, left ventricular ejection fraction.

**Figure 3:**
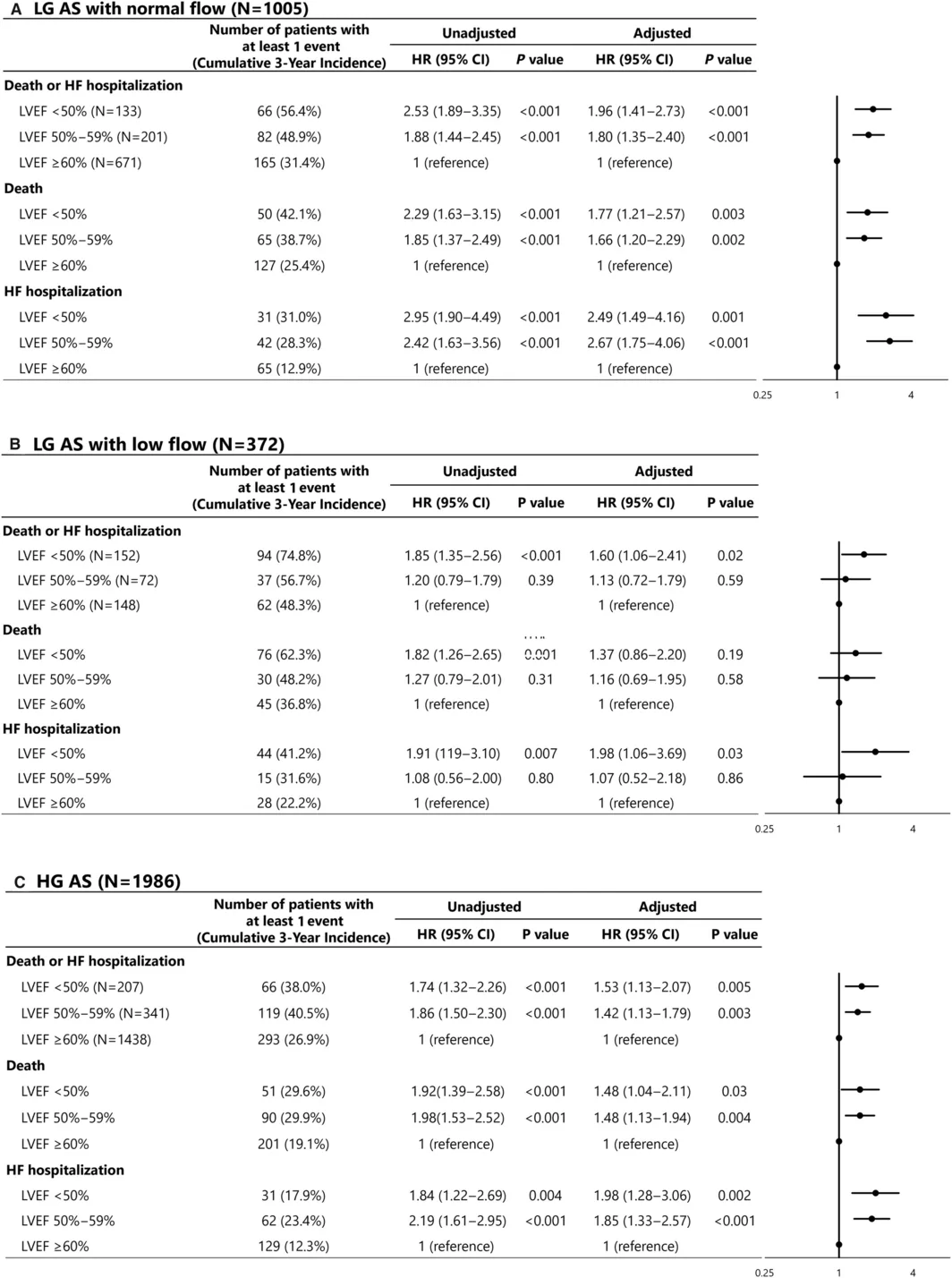
Crude and adjusted risks for clinical events according to the baseline left ventricular ejection fraction. **A**, LG AS with normal flow, **B**, LG with low flow, and **C**, HG AS. Cumulative incidence was estimated at 3 y, while the number of patients with event was counted throughout the entire follow‐up period. AS indicates aortic stenosis; HF, heart failure; HG, high gradient; HR, hazard ratio; LG, low gradient; and LVEF, left ventricular ejection fraction.

### Baseline Clinical Characteristics According to LVEF in LG AS With Low Flow

Among 372 patients with LG AS with low flow, patients with LVEF <50% compared with those with LVEF ≥60% had a higher prevalence of cardiovascular comorbidities including coronary artery disease and prior myocardial infarction (Table S1). Patients with LVEF <50% less often had atrial fibrillation compared with those with LVEF ≥60% (39% versus 57%). On the other hand, baseline characteristics were comparable between patients with LVEF 50% to 59% and those with LVEF ≥60% except for prior myocardial infarction and a history of coronary revascularization or HF. Patients with LVEF 50% to 59% had a high prevalence of atrial fibrillation/flutter, which was comparable with those with LVEF ≥60%. Echocardiographic parameters, including LV size and diastolic function, were comparable between patients with LVEF 50% to 59% and ≥60%. The AV calcification score by CT was comparable among the LVEF groups.

### Clinical Outcomes According to LVEF in LG AS With Low Flow

The cumulative 3‐year incidence of the primary outcome was significantly higher in the LVEF <50% group, but not in the LVEF 50% to 59% group, than in the LVEF ≥60% group (74.8%, 56.7%, and 48.3%, respectively) (Figures 2B and 3B). After adjusting for confounders, including the initial treatment strategies, the excess hazard of LVEF <50% relative to LVEF ≥60% (reference) remained significant for the primary outcome (HR, 1.60 [95% CI, 1.06–2.41], *P*=0.02), whereas the excess hazard of LVEF 50% to 59% was no longer significant for the primary outcome (HR, 1.13 [95% CI, 0.72–1.79], *P*=0.59) (Figure 3B). The excess hazard associated with LVEF <50% relative to LVEF ≥60% for the primary outcome in the conservative stratum was consistent with the main analysis, whereas it was not significant for the primary outcome in the initial AVR stratum (Figure S3).

### Baseline Clinical Characteristics According to LVEF in HG AS

Among 1986 patients with HG AS, patients with LVEF <50% and 50% to 59% compared with patients with LVEF ≥60% were more often men and had a higher prevalence of cardiovascular comorbidities and had greater frailty (Table S2). Patients with LVEF <50% and 50% to 59% had smaller AVA and stroke volume index, larger LV size and LV outflow tract diameter, and more frequent moderate‐to‐severe mitral regurgitation and pulmonary hypertension. The AV calcification score by CT was greater in the lower LVEF groups than in those with LVEF ≥60%.

### Clinical Outcomes According to LVEF in HG AS

The cumulative 3‐year incidence of the primary outcome was significantly higher in the low LVEF groups (<50% and 50%–59%) than in those with LVEF ≥60% (38.0%, 40.5%, and 26.9%, respectively) (Figures 2C and 3C). After adjusting for confounders, including the initial treatment strategy, LVEF <50% and 50% to 59% remained associated with greater hazards of the primary outcome compared with LVEF ≥60% (reference) (HR, 1.53[95% CI, 1.13–2.07], *P*=0.005; HR, 1.42 [95% CI, 1.13–1.79], *P*=0.003) (Figure 3C). The effect sizes relative to the reference group were numerically similar between the 2 groups of patients with LVEFs <50% and 50% to 59%. The greater hazards of the primary outcome associated with LVEF <50% and LVEF 50% to 59%, relative to LVEF ≥60%, were consistent with the main analysis in both the conservative and initial AVR strata (Figure S4). The association between lower LVEF and adverse outcomes was attenuated in the initial AVR stratum compared with the conservative stratum, although the effect of LVEF on clinical outcomes remained significant even in the initial AVR stratum (Figure S4).

### Sensitivity Analyses

The results of the sensitivity analyses in patients without prior myocardial infarction and in patients whose LVEF was measured using the biplane Simpson method of disks were fully consistent with the results in the entire study population (Figures S5 and S6).

## DISCUSSION

The main findings of the present study were as follows. (1) The greater hazard associated with LVEF <50% compared with LVEF ≥60% of a composite of death or HF hospitalization was consistently seen across the 3 hemodynamic subtypes of severe AS. (2) LVEF 50% to 59% was associated with similarly increased risk in LG AS with NF and HG AS but not in LG AS with low flow. In LG AS with NF and HG AS, the magnitude of excess risk of LVEF 50% to 59% relative to LVEF ≥60% was comparable to that of LVEFs <50% relative to LVEF ≥60%. (3) In stratified analyses according to initial treatment strategy, the association between lower LVEF and adverse outcomes was attenuated in the initial AVR stratum compared with the conservative stratum across all hemodynamic subtypes.

In HG AS, however, although the magnitude of the association was attenuated, the effect of LVEF on clinical outcomes remained significant even in the initial AVR stratum.

### Prognostic Implications of NFLG AS

NFLG AS (LG AS with NF and LVEF ≥50%) has traditionally been regarded as less severe and even equivalent to moderate AS in the 2025 European Society of Cardiology/European Association for Cardio‐Thoracic Surgery guidelines,^1^ largely based on small earlier studies with limited sample sizes. Tribouilloy et al. (n=85) and Chadha et al. (n=113) reported comparable outcomes between NFLG and moderate AS,^10^, ^11^ yet their cohorts predominantly included patients with preserved systolic function; median LVEF 65% (interquartile range, 60%–68% and 60%–69%, respectively). Mehrotra et al. (n=75) similarly excluded LVEF <55%, with a mean LVEF of 71.6±6.3%.^12^ Jander et al. examined a younger cohort (mean age 69.8 years, LVEF 66.9±5.7%) not fully representative of contemporary NFLG AS and with very few patients with LVEF 50% to 59%.^13^ Thus, prior studies could not adequately assess outcomes in patients with LVEF 50% to 59%.

In contrast, the present study is the first to stratify NFLG AS (n=872) by LVEF 50% to 59% (n=201) versus ≥60% (n=671), thereby revealing prognostic heterogeneity that earlier studies could not evaluate. We showed that patients with LVEF 50% to 59% had notably worse outcomes than those with LVEF ≥60%, with risk magnitude comparable to overt systolic dysfunction (LVEF <50%). By contrast, patients with LVEF ≥60% exhibited favorable prognosis, consistent with previous studies whose populations largely consisted of patients with LVEF ≥60%.^10^, ^11^, ^12^ These findings highlight that the prognosis of NFLG AS cannot be judged solely by flow and gradient criteria, and that even modest LVEF reduction identifies higher‐risk patients. Careful evaluation of LV systolic performance, beyond conventional hemodynamic classification, might therefore be essential to identify high‐risk patients within the NFLG AS subgroup. These results suggest that uniformly considering NFLG AS as equivalent to moderate AS might overlook a higher‐risk subset and warrants reconsideration of its clinical positioning.

### Redefining the Normal Threshold of LVEF in Severe AS

LVEF reflects both myocardial shortening and ventricular geometry, and systolic impairment may begin before overt LVEF reduction. Although current guidelines define LV dysfunction at LVEF <50%, recent guideline recommendations also suggest that clinically meaningful LV dysfunction may occur at higher LVEF thresholds. The 2020 American College of Cardiology/American Heart Association guidelines maintain 50% as the formal threshold for LV dysfunction, yet recommend consideration of AVR (Class IIb) in asymptomatic patients with high‐gradient severe AS if a progressive decrease in LVEF on at least 3 serial imaging studies to <60% was observed, acknowledging that even modest reductions in LVEF may carry prognostic concerns.^2^ Similarly, the 2025 European Society of Cardiology/European Association for Cardio‐Thoracic Surgery guidelines recommend intervention in asymptomatic severe AS as Class I when LVEF falls below 50%, and as Class IIa when LVEF is <55%, reflecting growing recognition that the “normal” range of LVEF may be higher than previously recognized. However, the prognostic implications of LVEF 50% to 59% across hemodynamic subtypes—and within treatment strata—have not been well defined.^14^ The present study strongly reinforced the evolving perspectives, demonstrating that LVEF 50% to 59% was associated with worse outcomes compared with LVEF ≥60%, with prognostic profiles similar to those with LVEF <50% in patients with LG AS with NF and HG AS. Furthermore, in stratified analyses according to initial treatment strategy, the association between lower LVEF and adverse outcomes appeared to be attenuated in the initial AVR stratum in patients with LG AS regardless of flow, whereas in HG AS, lower LVEF remained associated with worse outcomes even after AVR. These findings suggest that the prognostic impact of LVEF may be partially modified by intervention, while remaining clinically relevant in HG AS. In a prior mechanistic study, progressive impairment of LV systolic performance was observed once the AVA declined below ∼1.2 cm^2^, indicating that systolic dysfunction may already begin during the ostensibly “moderate” stage of AS.^4^ These findings raise the possibility that patients with LG AS with NF and LVEF 50% to 59% may represent a subgroup in whom myocardial contractile impairment has already begun despite preserved forward flow, potentially corresponding to an early stage in the transition from adaptive concentric remodeling to contractile failure. The present study demonstrated that stroke volume showed a gradual decline from LVEF ≥60% group to LVEF 50% to 59% and <50% group, even though flow remained within the “normal” range, suggesting a progressive impairment in LV systolic performance. The present findings might therefore capture patients at the inflection point where LV systolic decline is underway, implying that LG AS with NF and LVEF 50% to 59% already lies within the pathophysiologic spectrum of truly severe AS rather than merely “moderate” disease.

In contrast, no prognostic difference was observed between LVEF 50% to 59% and ≥60% in LG AS with low flow. This finding could be explained by the clinical characteristics of these 2 groups. The prevalence of atrial fibrillation was extremely high in both groups (69% in LVEF 50%–59% and 59% in LVEF ≥60%), suggesting that atrial fibrillation itself was a major contributor to the low‐flow state, regardless of modest reductions in LVEF. In addition, AS‐related symptoms were comparably frequent in these 2 groups, supporting the notion that their overall disease burden was similar, although prior myocardial infarction was more prevalent in the LVEF 50% to 59% group. By contrast, patients with LVEF <50% showed a different risk profile, characterized by a lower prevalence of atrial fibrillation (39%) but a markedly higher rate of HF hospitalization at the time of index echocardiography (37%), together with more frequent AS‐related symptoms, greater LV enlargement, and more prevalent mitral regurgitation. These features likely reflect the combined burden of reduced EF and impaired forward flow, which translated into significantly worse outcomes in this group. We did not detect a clear prognostic separation between patients with LVEF 50% to 59% and those with LVEF ≥60%, although the modest sample size of the 50% to 59% subgroup limits precision. This observation might provide empirical support for the current definition of paradoxical low‐flow LG AS, which combines patients with preserved (≥50%) LVEF into a single clinical entity irrespective of whether it is in the 50% to 59% or ≥60% range.

### Study Limitations

This study had several limitations. First, the echocardiographic data were site reported, introducing the possibility of measurement variability. AVA calculation is particularly sensitive to LV outflow tract diameter measurements, and overestimation could lead to misclassification of AS subtypes. However, in the present study, mean LV outflow tract diameters were ∼20 mm across groups, within expected physiological ranges, and training workshops were conducted to minimize measurement variability. Because a central echocardiography core laboratory was not used, we could not formally assess interobserver reproducibility or perform coefficient of variation analyses for key echocardiographic variables, which remains an important limitation of the present registry analysis. The absence of a central core laboratory remains an important limitation for reproducibility of LVEF measurement, particularly when dealing with narrow categories such as 50% to 59% versus ≥60%. Second, some LVEF measurements used the Teichholz method, which may be vulnerable to measurement variability. However, sensitivity analyses restricted to patients whose LVEF was measured using the biplane Simpson method yielded results fully consistent with the primary analysis. Third, the prognostic impact of AVR was not directly evaluated, and serial changes in LVEF after intervention were not systematically analyzed. Although initial treatment strategy was included in risk adjustment and stratified analyses were performed in the initial AVR and conservative strata, dedicated studies are warranted to clarify the effect of AVR on ventricular remodeling and outcomes in these subgroups. A major unmet need remains the lack of randomized trials evaluating AVR in paradoxical low flow LG and NFLG AS. Prior observational comparisons of AVR versus conservative management have also been limited by selection bias (including patients with contraindications) and, in some studies, limited incorporation of transcatheter AV implantation.^15^ Further studies are warranted to evaluate the benefit of initial AVR in the 3 subtypes of LG AS. Fourth, CT was not systematically acquired or analyzed for CT‐derived AVA planimetry across all patients; therefore, we cannot provide a direct echo AVA versus CT AVA comparison in the overall cohort. Finally, the results should be interpreted with caution due to the nonrandomized nature of the data. Because patients with lower LVEF had more cardiovascular comorbidity, particularly in the LG AS with NF subgroup, residual confounding remains possible despite multivariable adjustment.

## CONCLUSIONS

LVEF 50% to 59% compared with LVEF ≥60% was consistently associated with worse clinical outcomes in patients with HG AS and LG AS with NF, with risks comparable to those with LVEF <50%. These findings suggest that LVEF may provide additional prognostic stratification within severe AS phenotypes, particularly in NFLG AS, and suggest that an LVEF threshold of 60% may better identify a lower‐risk subgroup.

## Sources of Funding

This work was supported by an educational grant from the Research Institute for Production Development (Kyoto, Japan).

## Disclosures

Tomohiko Taniguchi: lecture fees from Edwards Lifesciences, Medtronic, and Abbott. Shinichi Shirai: clinical proctors for Edwards Lifesciences, Medtronic, and Abbott. No other disclosures were reported.

## Supporting information

Appendix S1: List of Investigators
Supplementary Methods: Data S1 and S2
Tables S1–S2
Figures S1–S6

STROBE Statement
