# Supplementary material for: Prognostic Implications of Left Ventricular Ejection Fraction Across the Flow‐Gradient Patterns in Severe Aortic Stenosis
Source: J Am Heart Assoc. 2026 Sep 24;15(19):e050412. doi: 10.1161/JAHA.126.050412 (PMC13745010; doi:10.1161/JAHA.126.050412)
Supplement: Supplementary file 1 [file JAH3-15-e050412-s001.pdf]

STROBE Statement—checklist of items that should be included in reports of observational studies

	Item No.	Recommendation	Page No.	Relevant text from manuscript
**Title and abstract**	1	(*a*) Indicate the study's design with a commonly used term in the title or the abstract	2	consecutive patients with severe AS from the CURRENT AS Registry-2
		(*b*) Provide in the abstract an informative and balanced summary of what was done and what was found	2	consecutive patients with severe AS from the CURRENT AS Registry-2
**Introduction**				
Background/rationale	2	Explain the scientific background and rationale for the investigation being reported	5	the subset with preserved left ventricular ejection fraction (LVEF) and normal flow (NF), which is referred to as NFLG AS, is regarded as "unlikely to be severe AS" even though it met the hemodynamic criteria for severe AS with an aortic valve area (AVA) <1 cm2.
Objectives	3	State specific objectives, including any prespecified hypotheses	5	Therefore, we compared clinical and echocardiographic characteristics as well as clinical outcomes across LVEF categories (<50%, 50-59%, and ≥60%) within each hemodynamic subtype (LG AS with normal flow, LG AS with low flow and high-gradient [HG] AS) in a large Japanese database of consecutive patients

				with severe AS.
**Methods**				
Study design	4	Present key elements of study design early in the paper	6	The CURRENT AS (Contemporary outcomes after sURgery and medical tREatmeNT in patients with severe Aortic Stenosis) Registry-2 is a prospective multi-center registry conducted among 21 participating centers in Japan (Supplemental Appendix A). The registry design and enrollment procedures have been described previously. Briefly, 3369 consecutive patients were enrolled between April 2018 and December 2020 when they were first identified as having severe AS, defined by at least 1 of the following echocardiographic criteria: peak aortic jet velocity (Vmax) >4.0 m/s, mean aortic pressure gradient (PG) >40 mmHg, or aortic valve area (AVA) <1.0 cm^2.

Setting	5	Describe the setting, locations, and relevant dates, including periods of recruitment, exposure, follow-up, and data collection	6-8	The CURRENT AS (Contemporary outcomes after sURgery and medical tREatmeNT in patients with severe Aortic Stenosis) Registry-2 is a prospective multi-center registry conducted among 21 participating centers in Japan (Supplemental Appendix A). The registry design and enrollment procedures have been described previously. Briefly, 3369 consecutive patients were enrolled between April 2018 and December 2020 when they were first identified as having severe AS, defined by at least 1 of the following echocardiographic criteria: peak aortic jet velocity (Vmax) >4.0 m/s, mean aortic pressure gradient (PG) >40 mmHg, or aortic valve area (AVA) <1.0 cm^2.
Participants	6	(*a*) *Cohort study*—Give the eligibility criteria, and the sources and methods of selection of participants. Describe methods of follow-up<br>*Case-control study*—Give the eligibility criteria, and the sources and methods of case	6-7	The CURRENT AS (Contemporary outcomes after sURgery and medical

		ascertainment and control selection. Give the rationale for the choice of cases and controls<br>*Cross-sectional study*—Give the eligibility criteria, and the sources and methods of selection of participants		tREatmeNT in patients with severe Aortic Stenosis) Registry-2 is a prospective multi-center registry conducted among 21 participating centers in Japan (Supplemental Appendix A). The registry design and enrollment procedures have been described previously. Briefly, 3369 consecutive patients were enrolled between April 2018 and December 2020 when they were first identified as having severe AS, defined by at least 1 of the following echocardiographic criteria: peak aortic jet velocity (Vmax) >4.0 m/s, mean aortic pressure gradient (PG) >40 mmHg, or aortic valve area (AVA) <1.0 cm^2.
		(*b*) *Cohort study*—For matched studies, give matching criteria and number of exposed and unexposed<br>*Case-control study*—For matched studies, give matching criteria and the number of controls per case		
Variables	7	Clearly define all outcomes, exposures, predictors, potential confounders, and effect modifiers. Give diagnostic criteria, if applicable	8-9	The primary outcome measure for this analysis

				was a composite of all-cause death or heart failure (HF) hospitalization.
Data sources/ measurement	8*	For each variable of interest, give sources of data and details of methods of assessment (measurement). Describe comparability of assessment methods if there is more than one group	*7-9*	All participants underwent a comprehensive 2-dimensional and Doppler echocardiographic evaluation at each participating center.
Bias	9	Describe any efforts to address potential sources of bias	8-9	As an exploratory analysis, we evaluated the effects of LVEF on clinical outcomes in patients without prior myocardial infarction and in patients whose LVEFs were measured using the biplane Simpson method of disks.
Study size	10	Explain how the study size was arrived at	6	The CURRENT AS (Contemporary outcomes after sURgery and medical tREatmeNT in patients with severe Aortic Stenosis) Registry-2 is a prospective multi-center registry conducted among 21 participating centers in Japan (Supplemental Appendix A). The registry design and enrollment

	procedures have been described previously. Briefly, 3369 consecutive patients were enrolled between April 2018 and December 2020 when they were first identified as having severe AS, defined by at least 1 of the following echocardiographic criteria: peak aortic jet velocity (Vmax) >4.0 m/s, mean aortic pressure gradient (PG) >40 mmHg, or aortic valve area (AVA) <1.0 cm^2.

Continued on next page

Quantitative variables	11	Explain how quantitative variables were handled in the analyses. If applicable, describe which groupings were chosen and why	7	The resulting 3363 patients were categorized into the 3 hemodynamic strata according to baseline flow and gradient (Figure 1): LG AS with normal flow, defined as Vmax <4 m/s, mean aortic PG <40 mmHg, AVA <1 cm^2, and indexed stroke volume ≥35 mL/m^2 (N=1005); LG AS with low flow, defined as Vmax <4 m/s, mean aortic PG <40 mmHg, AVA <1 cm^2, and indexed stroke volume <35 mL/m^2 (N=372); HG AS, defined as Vmax ≥4 m/s or mean PG ≥40 mmHg (N=1986) (Figure 1).
Statistical methods	12	(*a*) Describe all statistical methods, including those used to control for confounding	9	In the multivariable Cox proportional hazard models, we used 22 clinically relevant covariates listed in Table 1 including the initial treatment strategy as the risk-adjusting variables and the centers were incorporated as the stratification variable, consistent with our previous report.
		(*b*) Describe any methods used to examine subgroups and interactions	8-9	Because the primary objective was to characterize

			the prognostic implications of LVEF within individual hemodynamic phenotypes, the primary analyses did not include interaction testing between LVEF category and AS subtype.
	(*c*) Explain how missing data were addressed	6-9	For the present study, 6 of the 3369 registered patients were excluded: 1 because baseline LVEF was unavailable and 5 because stroke volume index was unavailable in the setting of LG AS.<br>There were no missing data other than body mass index among the risk-adjusting variables, and the missing data in body mass index were in 11 patients in the present study population. Because we considered the effect of missing was minor, we constructed the multivariable models with those without missing data.
	(*d*) *Cohort study*—If applicable, explain how loss to follow-up was addressed<br>*Case-control study*—If applicable, explain how matching of cases and controls was addressed<br>*Cross-sectional study*—If applicable, describe analytical methods taking account of sampling strategy	9	Cumulative incidence was estimated by the Kaplan-Meier method and the differences among LVEF <50%, LVEF 50-59% and LVEF ≥60% (reference) were

				assessed by the log-rank test within each of the 3 hemodynamic subtypes of AS.
		(*e*) Describe any sensitivity analyses	9	As an exploratory analysis, we evaluated the effects of LVEF on clinical outcomes in patients without prior myocardial infarction and in patients whose LVEFs were measured using the biplane Simpson method of disks.
**Results**				
Participants	13*	(a) Report numbers of individuals at each stage of study—eg numbers potentially eligible, examined for eligibility, confirmed eligible, included in the study, completing follow-up, and analysed	10	The resulting 3363 patients were categorized into the 3 hemodynamic strata according to baseline flow and gradient (Figure 1): LG AS with normal flow, defined as Vmax <4 m/s, mean aortic PG <40 mmHg, AVA <1 cm^2, and indexed stroke volume ≥35 mL/m^2 (N=1005); LG AS with low flow, defined as Vmax <4 m/s, mean aortic PG <40 mmHg, AVA <1 cm^2, and indexed stroke volume <35 mL/m^2 (N=372); HG AS, defined as Vmax ≥4 m/s or mean PG ≥40 mmHg

				(N=1986) (Figure 1).
		(b) Give reasons for non-participation at each stage	6-7, 30	For the present study, 6 of the 3369 registered patients were excluded: 1 because baseline LVEF was unavailable and 5 because stroke volume index was unavailable in the setting of LG AS.
		(c) Consider use of a flow diagram	30	Figure 1
Descriptive data	14*	(a) Give characteristics of study participants (eg demographic, clinical, social) and information on exposures and potential confounders	10	Among 1005 patients with LG AS with normal flow, baseline characteristics were significantly different across the LVEF groups (Table 1).
		(b) Indicate number of participants with missing data for each variable of interest	26-28	Table 1
		(c) *Cohort study*—Summarise follow-up time (eg, average and total amount)	10	The median follow-up period in the entire cohort was 763 (IQR: 426-1059) days.
Outcome data	15*	*Cohort study*—Report numbers of outcome events or summary measures over time	*10-11*	*The cumulative 3-year incidence of the primary outcome was significantly higher in the low LVEF groups (<50% and 50-59%) than in the LVEF ≥60% group (56.4%, 48.9%, and 31.4%, respectively) (Figure 2A and 3A).*
		Case-control study—Report numbers in each exposure category, or summary measures of exposure		
		Cross-sectional study—Report numbers of outcome events or summary measures		
Main results	16	(*a*) Give unadjusted estimates and, if applicable, confounder-adjusted estimates and their precision (eg, 95% confidence interval). Make clear which confounders were adjusted for and why they were included	10-11	After adjusting confounders including the initial treatment strategies, the excess risks of LVEF <50% and 50-59%, respectively,

			relative to LVEF ≥60% (reference) remained significant for the primary outcome (HR 1.96, 95% CI: 1.41-2.73, P<0.001; HR 1.80, 95% CI: 1.35-2.40, P<0.001) as well as for the individual components of the primary outcome (Figure 3A).
	(*b*) Report category boundaries when continuous variables were categorized	11, 26-28	Table 1
	(*c*) If relevant, consider translating estimates of relative risk into absolute risk for a meaningful time period	11	The cumulative 3-year incidence of the primary outcome was significantly higher in the low LVEF groups (<50% and 50-59%) than in the LVEF ≥60% group (56.4%, 48.9%, and 31.4%, respectively) (Figure 2A and 3A). After adjusting confounders including the initial treatment strategies, the excess risks of LVEF <50% and 50-59%, respectively, relative to LVEF ≥60% (reference) remained significant for the primary outcome (HR 1.96, 95% CI: 1.41-2.73, P<0.001; HR 1.80, 95% CI: 1.35-2.40, P<0.001) as well as for the individual components of the primary outcome (Figure 3A).

Continued on next page

Other analyses	17	Report other analyses done—eg analyses of subgroups and interactions, and sensitivity analyses	12-13	The results of the sensitivity analyses in patients without prior myocardial infarction and in patients whose LVEFs were measured using the biplane Simpson method of disks were fully consistent with the results in the entire study population (Supplementary Figure 5 and 6).
**Discussion**				
Key results	18	Summarise key results with reference to study objectives	13-14	The main findings of the present study were as follows: (1) The higher risk of LVEF <50% compared to LVEF ≥60% for a composite of death or HF hospitalization was consistently seen across the 3 hemodynamic subtypes of severe AS; (2) LVEF 50-59% was associated with similarly increased risk in LG AS with normal flow and HG AS, but not in LG AS with low flow. In LG AS with normal flow and HG AS, the magnitude of excess risk of LVEF 50-59% relative to LVEF ≥60% was comparable to that of LVEFs <50% relative to LVEF ≥60%; (3) In stratified analyses according to initial treatment strategy, the association between lower LVEF and adverse outcomes

				was attenuated in the initial AVR stratum compared with the conservative stratum across all hemodynamic subtypes.<br>In HG AS, however, although the magnitude of the association was attenuated, the effect of LVEF on clinical outcomes remained significant even in the initial AVR stratum.
Limitations	19	Discuss limitations of the study, taking into account sources of potential bias or imprecision. Discuss both direction and magnitude of any potential bias	18	This study had several limitations. First, the echocardiographic data were site-reported, introducing the possibility of measurement variability.
Interpretation	20	Give a cautious overall interpretation of results considering objectives, limitations, multiplicity of analyses, results from similar studies, and other relevant evidence	14-18	In contrast, the present study is the first to stratify NFLG AS (n=872) by LVEF 50–59% (n=201) versus ≥60% (n=671), thereby revealing prognostic heterogeneity that earlier studies could not evaluate.
Generalisability	21	Discuss the generalisability (external validity) of the study results	14-18	These results suggest that uniformly considering NFLG AS as equivalent to moderate AS might overlook a higher-risk subset and warrants reconsideration of its clinical positioning.
**Other information**				
Funding	22	Give the source of funding and the role of the funders for the present study and, if applicable, for the original study on which the present article is based	20	Funding: This work was supported by an educational grant from the Research Institute for Production

Development (Kyoto, Japan).

*Give information separately for cases and controls in case-control studies and, if applicable, for exposed and unexposed groups in cohort and cross-sectional studies.

Note: An Explanation and Elaboration article discusses each checklist item and gives methodological background and published examples of transparent reporting. The STROBE checklist is best used in conjunction with this article (freely available on the Web sites of PLoS Medicine at http://www.plosmedicine.org/, Annals of Internal Medicine at http://www.annals.org/, and Epidemiology at http://www.epidem.com/). Information on the STROBE Initiative is available at www.strobe-statement.org.
