# Supplementary material for: Prognostic Implications of Left Ventricular Ejection Fraction Across the Flow‐Gradient Patterns in Severe Aortic Stenosis
Source: J Am Heart Assoc. 2026 Sep 24;15(19):e050412. doi: 10.1161/JAHA.126.050412 (PMC13745010; doi:10.1161/JAHA.126.050412)
Supplement: Supplementary file 2 [file JAH3-15-e050412-s002.pdf]

Supplemental Material

Appendix: List of participating centers and investigators

Cardiology

Kyoto University Hospital: Takeshi Kimura, Naritatsu Saito, Takao Kato, Hirotoshi Watanabe, Hiroki Shiomi, Toshiaki Toyota, Eri Minamino-Muta, Yasauki Takeji, Tomoya Yoneda, Nao Kaneko, Yuki Obayashi, Ko Yamamoto

Kokura Memorial Hospital: Kenji Ando, Shinichi Shirai, Tomohiko Taniguchi, Hiroyuki Tabata, Kazuki Kitano

Kobe City Medical Center General Hospital: Yutaka Furukawa, Takeshi Kitai

National Cerebral and Cardiovascular Center: Chisato Izumi, Masashi Amano

Tenri Hospital: Makoto Miyake

Kansai Electric Power Hospital: Katsuhisa Ishii, Shunsuke Usami

Osaka Red Cross Hospital: Tsukasa Inada, Kazuya Nagao

Kitano Hospital: Moriaki Inoko

Japanese Red Cross Wakayama Medical Center: Takashi Tamura, Mamoru Toyofuku

Mitsubishi Kyoto Hospital: Shinji Miki, Takashi Yokomatsu, Masashi Kato, Kenji Nakatsuma

Kurashiki Central Hospital: Kazushige Kadota, Ryosuke Murai, Kohei Osakada

Shiga General Hospital: Shigeru Ikeguchi, Yasutaka Inuzuka

Shimada General Medical Center: Takeshi Aoyama, Norio Kanamori

Shizuoka City Shizuoka Hospital: Tomoya Onodera, Koichiro Murata

Shizuoka General Hospital: Hiroki Sakamoto, Yasuyo Takeuchi, Tomohisa Tada

Kindai University Nara Hospital: Manabu Shirotani

Kishiwada City Hospital: Mitsuo Matsuda, Masayasu Izuhara

Hyogo Prefectural Amagasaki General Medical Center: Yukihito Sato, Hiroyuki Nakayama

National Hospital Organization Kyoto Medical Center: Masaharu Akao, Mitsuru Ishii

Koto Memorial Hospital: Hiroshi Mabuchi, Masahiro Kimura

Japanese Red Cross Otsu Hospital: Kazuaki Kaitani, Fumiko Nakazeki, Marie Okabayashi

Cardiovascular surgery

Kyoto University Hospital: Kenji Minatoya, Kazuhiro Yamazaki

Kokura Memorial Hospital: Nobuhisa Ohno, Yoshio Arai

Kobe City Medical Center General Hospital: Tadaaki Koyama

National Cerebral and Cardiovascular Center: Tomoyuki Fujita, Satsuki Fukushima

Tenri Hospital: Atsushi Iwakura, Kyokun Uehara

Kansai Electric Power Hospital: Etsuro Suenaga

Osaka Red Cross Hospital: Shogo Nakayama, Michihito Nonaka

Kitano Hospital: Michiya Hanyu

Japanese Red Cross Wakayama Medical Center: Naoki Kanemitsu, Hisashi Sakaguchi

Mitsubishi Kyoto Hospital: Hitoshi Okabayashi, Jiro Esaki

Kurashiki Central Hospital: Tatsuhiko Komiya, Jota Nakano, Shingo Hirao

Shiga General Hospital: Yamada Tomoyuki, Kazuhiko Katsuyama

Shizuoka City Shizuoka Hospital: Fumio Yamazaki, Hiroshi Mitsuoka, Masanao Nakai

Shizuoka General Hospital: Hiroshi Tsuneyoshi

Kindai University Nara Hospital: Noboru Nishiwaki

Kishiwada City Hospital: Onoe Nasahiko, Kosuke Fujii

Hyogo Prefectural Amagasaki General Medical Center: Nobushige Tamura, OkadaTatsuji

National Hospital Organization Kyoto Medical Center: Kotaro Shiraga, Tsuyoshi Kataoka

Koto Memorial Hospital: Toshihiro Kawahira, Fumihiro Miyashita

Japanese Red Cross Otsu Hospital: Kouji Ueyama

Principal Investigator (Cardiovascular surgery)：Kenji Minatoya

Principal Investigator (Cardiology)：Takeshi Kimura

Supplemental Methods

Data S1: Echocardiographic and computed tomographic evaluation

All participants underwent a comprehensive 2-dimensional and Doppler echocardiographic evaluation at each participating center. Vmax and mean aortic PG measurements were obtained with the use of the simplified Bernoulli equation. AVA was calculated using the standard continuity equation, and indexed to body surface area. We made every effort to standardize the quality of echocardiography when assessing the severity of AS by holding a workshop or a hands-on seminar to provide training for the echo technicians in the participating centers. In each participating center, we highly recommended the use of multiple acoustic windows (apical, high parasternal or right parasternal) to obtain the greatest peak velocity in accordance with the guidelines. Vmax was defined as the highest velocity obtained from any window after careful examination. Averaging of several beats was mandatory with irregular rhythms. Special care was taken to select representative sequences of beats and to avoid post-extrasystolic beats. Stroke volume was calculated by multiplying the cross-sectional left ventricular outflow tract (LVOT) area with LVOT velocity-time integral (VTI). The left ventricular (LV) end-diastolic volume, LV end-systolic volume, and left ventricular ejection fraction (LVEF) were primarily measured using the biplane Simpson method, with use of the Teichholz method in some patients. We recommended dobutamine stress echocardiography in patients with LG AS with reduced LVEF to assess whether they had true severe AS in accordance with the guidelines. According to the extent of cardiac damage on echocardiography, patients were classified as stage 0 (no extra-aortic valve cardiac damage), stage 1 (LV damage as defined as the presence of LV hypertrophy [LV mass index >95 g/m^2 in women and >115 g/m^2 in men], and/or elevated LV filling pressures [a lateral E/e' ratio >13 or a septal E/e' ratio >15], and/or LV systolic dysfunction [LVEF <50%]); Stage 2 (left atrial [LA] and/or mitral valve damage as defined by the presence of LA enlargement [LA anteroposterior

diameter >40 mm in the parasternal long-axis view], and/or atrial fibrillation, and/or moderate or severe mitral regurgitation [MR]); Stage 3-4 (pulmonary vasculature and/or tricuspid valve damage as defined by the presence of systolic pulmonary hypertension [systolic pulmonary arterial pressure ≥60 mmHg], and/or the presence of moderate or severe tricuspid regurgitation [TR], and/or right ventricular [RV] damage as defined by the presence of moderate or greater RV systolic dysfunction) on the basis of the recently proposed cardiac damage staging scheme. If patients met the criteria for multiple stages, they were assigned to the highest (worst) stage. LV mass was calculated with the formula recommended by the American Society of Echocardiography and was indexed to the body surface area as follows: LV mass=0.8×1.04 [LV end-diastolic diameter + PWT in diastole + IVST in diastole]^3− (LV end-diastolic diameter)^3] + 0.6.

Relative wall thickness (RWT) was calculated with the formula: (2 × PWT in diastole) / LV end-diastolic diameter.

The echocardiographic variables that define LV geometry (LV mass index and RWT) were subsequently used to categorize patients into four patterns of LV remodeling: normal geometry (normal LV mass index and RWT ≤0.42), concentric remodeling (normal LV mass index and RWT >0.42), concentric hypertrophy (high LV mass index and RWT >0.42), and eccentric hypertrophy (high LV mass index and RWT ≤0.42).

We recommended CT imaging including an electrocardiography (ECG)-gated non-contrast scan with slice thickness of less than or equal to 3 mm and an ECG-gated contrast enhanced scan with multiple phases reconstructed during systole and diastole to evaluate the aortic valve calcification, aortic annulus, aorta, and transfemoral access. The region of interest to determine aortic valve calcification included the aortic valve leaflets and the virtual annulus defined by the leaflet hinge points. Calcifications in the aortic sinus, coronary arteries, left

ventricular outflow tract, sino-tubular junction and mitral annulus were excluded after confirmation of the aortic annulus, which was manually traced by selecting the nadir of the 3 cusps. The images were analyzed with the 3mensio Structural Heart 10.4 (Pie Medical Imaging, Maastricht, the Netherlands).

Data S2: Definitions of the clinical events

Death was regarded as cardiovascular in origin unless obvious non-cardiovascular causes could be identified. Sudden death was defined as death that occurred suddenly and unexpectedly in previously stable patients, in whom specific causes of sudden death were not identified other than severe AS. Death was not judged as sudden death if the death was confirmed to be due to other causes such as myocardial infarction or acute aortic dissection. Any death during the hospitalization for aortic valve replacement or transcatheter aortic valve implantation was regarded as aortic valve procedure-related death. Stroke was defined as duration of a focal or global neurological deficit ≥24 h; OR <24 h if available neuroimaging documents a new hemorrhage or infarct; OR the neurological deficit results in death. Ischemic stroke was defined as an acute episode of focal cerebral, spinal, or retinal dysfunction caused by infarction of the central nervous system tissue. Hemorrhagic stroke was defined as an acute episode of focal or global cerebral or spinal dysfunction caused by intraparenchymal, intraventricular, or subarachnoid hemorrhage. Disabling stroke was defined as a modified Rankin Scale (mRS) score of 2 or more at 90 days and an increase in at least one mRS category from an individual's pre-stroke baseline. Non-disabling stroke was defined an mRS score of <2 at 90 days or one that does not result in an increase in at least one mRS category from an individual's pre-stroke baseline. HF hospitalization was defined as hospitalization due to worsening HF requiring intravenous drug therapy. The Valve Academic Research Consortium (VARC)- 3 bleeding was defined according to Bleeding Academic Research Consortium (BARC) definition. Major bleeding was defined Type 2-4 bleeding in VARC-3 in this study. The causes of death and the clinical events were classified according to the Valve Academic Research Consortium definitions and were adjudicated by a clinical event committee.

Overt bleeding was defined that fulfils one of the following criteria:

Type 1

- Overt bleeding that does not require surgical or percutaneous intervention, but does require medical intervention by a health care professional, leading to hospitalization, an increased level of care, or medical evaluation (BARC 2)
- Overt bleeding that requires a transfusion of 1 unit* of whole blood/red blood cells (BARC 3a)

Type 2

- Overt bleeding that requires a transfusion of 2–4 units of whole blood/red blood cells (BARC 3a)
- Overt bleeding associated with a hemoglobin drop of >3 g/dL (>1.86 mmol/L) but <5 g/d (<3.1 mmol/L) (BARC 3a)

Type 3

- Overt bleeding in a critical organ, such as intracranial, intraspinal, intraocular, pericardial (associated with hemodynamic compromise/tamponade and necessitating intervention), or intramuscular with compartment syndrome (BARC 3b, BARC 3c)
- Overt bleeding causing hypovolemic shock or severe hypotension (systolic blood pressure <90 mmHg lasting >30 min and not responding to volume resuscitation) or requiring vasopressors or surgery (BARC 3b)
- Overt bleeding requiring reoperation, surgical exploration, or re-intervention for the purpose of controlling bleeding (BARC 3b, BARC 4)
- Post-thoracotomy chest tube output ≥2 L within a 24-h period (BARC 4)
- Overt bleeding requiring a transfusion of ≥5 units of whole blood/red blood cells (BARC 3a)
- Overt bleeding associated with a hemoglobin drop ≥5 g/dL (≥3.1 mmol/L) (BARC 3b).

Type 4

- Overt bleeding leading to death (BARC 5).

*Units of blood transfusion are described as Japanese unit.

List of the clinical event committee members

Hiroki Shiomi (Kyoto University Hospital), Hirotoshi Watanabe (Kyoto University Hospital), Kenji Nakatsuma (Mitsubishi Kyoto Hospital)

Table S1. Baseline clinical and echocardiographic characteristics according to the baseline left ventricular ejection fraction: LG AS with Low Flow.

LG AS with Low Flow	LVEF <50%	LVEF 50-59%	LVEF ≥60% (reference)			Missing value
	N=152	N=72	N=148	LVEF <50% versus LVEF ≥60%	LVEF 50-59% versus LVEF ≥60%	
**Clinical characteristics**						
Age, years [a]	81.6±9.7	82.2±9.3	82.6±7.5	0.32	0.68	0
Men [a]	94 (62%)	32 (44%)	59 (40%)	<0.001	0.52	0
BMI, kg/m^2 [a]	22.0±3.4	22.6±3.6	23.5±4.8	0.002	0.17	0
Hypertension [a]	122 (80%)	57 (79%)	117 (79%)	0.79	0.98	0
Current smoking [a]	5 (3%)	5 (7%)	1 (0.7%)	0.11	0.007	0
Diabetes	76 (50%)	25 (35%)	45 (30%)	<0.001	0.52	0
On insulin therapy [a]	19 (13%)	3 (4%)	3 (2%)	<0.001	0.36	0
Prior myocardial infarction [a]	33 (22%)	10 (14%)	6 (4%)	<0.001	0.008	0
Coronary artery disease [a]	97 (64%)	29 (40%)	46 (31%)	<0.001	0.18	0
Prior symptomatic stroke [a]	36 (24%)	11 (15%)	24 (16%)	0.11	0.86	0
Atrial fibrillation or flutter [a]	59 (39%)	50 (69%)	84 (57%)	0.002	0.07	0
Aortic/peripheral vascular disease [a]	22 (14%)	5 (7%)	19 (13%)	0.68	0.19	0
Creatinine level >2 mg/dl and non-dialysis [a]	12 (8%)	3 (4%)	6 (4%)	0.16	0.97	0
Dialysis [a]	20 (13%)	10 (14%)	14 (9%)	0.31	0.32	0
Anemia [a, b]	97 (64%)	27 (38%)	60 (41%)	<0.001	0.67	0
Liver cirrhosis (Child-Pugh B/C) [a]	3 (2%)	0 (0%)	1 (0.7%)	0.33	0.48	0
Malignancy currently under treatment [a]	15 (10%)	3 (4%)	4 (3%)	0.01	0.56	0
Chronic lung disease (moderate or severe) [a]	11 (7%)	2 (3%)	9 (6%)	0.69	0.29	0
STS score (PROM), %	6.1 (3.9-9.5)	5.0 (3.2-7.6)	4.1 (2.6-6.4)	<0.001	0.054	0
Symptoms possibly related to AS	126 (83%)	40 (56%)	84 (57%)	<0.001	0.87	0

HF hospitalization at the index echocardiography [a]	56 (37%)	12 (17%)	12 (8%)	<0.001	0.06	0
Clinical frailty scale [a, c] 1-3	38 (25%)	28 (39%)	52 (35%)	0.15	0.80	0
4-6	89 (59%)	34 (47%)	77 (52%)			
7-9	25 (16%)	10 (14%)	19 (13%)			
	N=122	N=49	N=103			98
BNP, pg/ml	638 (303-1436)	258 (121-540)	161 (61-331)	<0.001	0.006	
	N=20	N=8	N=21			323
NT-proBNP, pg/ml	2967 (2038-17919)	1471 (682-4369)	1567 (906-3344)	0.01	0.77	
**Etiology of aortic stenosis**						
Degenerative	147 (97%)	70 (97%)	138 (93%)	0.09	0.37	0
Congenital (unicuspid, bicuspid, or quadricuspid)	4 (3%)	0	3 (2%)			
Rheumatic	1 (0.7%)	2 (3%)	7 (5%)			
**Echocardiographic variables**						
Vmax, m/s	2.96±0.52	3.09±0.52	3.18±0.47	<0.001	0.21	0
Mean aortic PG, mm Hg	20.6±7.6	22.1±8.2	22.5±7.4	0.027	0.73	1
AVA (equation of continuity), cm^2	0.74±0.15	0.71±0.18	0.73±0.14	0.67	0.52	0
Indexed AVA, cm^2/m^2	0.48±0.11	0.47±0.11	0.47±0.09	0.47	0.66	0
LV end-diastolic diameter, mm	51.5±7.8	43.8±6.6	42.4±5.3	<0.001	0.08	0
LV end-systolic diameter, mm	41.9±8.5	30.5±5.9	26.8±4.3	<0.001	<0.001	1
LVEF, %	36±9	55±3	66±5	<0.001	<0.001	0
High LV mass index (men >115 g/m^2, and women >95g/m^2)	109 (72%)	28 (39%)	39 (26%)	<0.001	0.06	0
Relative wall thickness >0.42	48 (32%)	46 (64%)	102 (64%)	<0.001	0.46	0
LV remodeling pattern						
Concentric hypertrophy	37 (24%)	18 (25%)	29 (20%)	<0.001	0.20	0
Concentric remodeling	11 (7%)	28 (39%)	73 (49%)			
Eccentric hypertrophy	72 (47%)	10 (14%)	10 (7%)			
Normal	32 (21%)	16 (22%)	36 (24%)			
LVOT, mm	20.6±2.0	20.1±1.9	19.4±1.9	<0.001	0.007	0
SV index, ml/m^2	28±5	28±5	30±4	<0.001	0.001	0
Septal E/e'>15	107/131 (82%)	40/60 (67%)	80/122 (66%)	0.004	0.88	59
Cardiac damage						

Stage 0	0	2 (3%)	12 (8%)	<0.001	0.42	0
Stage 1	25 (16%)	5 (7%)	12 (8%)			
Stage 2	93 (61%)	44 (61%)	89 (60%)			
Stage 3-4	34 (22%)	21 (29%)	35 (24%)			
Any combined valvular disease (moderate or severe) [a]	58 (38%)	28 (39%)	52 (35%)	0.59	0.59	0
Moderate or severe AR	10 (7%)	2 (3%)	10 (7%)	0.95	0.22	0
Moderate or severe MS	5 (3%)	3 (4%)	8 (5%)	0.37	0.69	0
Moderate or severe MR	44 (29%)	14 (19%)	23 (16%)	0.005	0.47	0
Moderate or severe TR	27 (18%)	21 (29%)	30 (20%)	0.58	0.14	0
TR pressure gradient ≥40 mm Hg [a]	36 (24%)	16 (22%)	21 (14%)	0.04	0.14	0
**CT variables**	N=50	N=19	N=43			
Agatston score of aortic valve, AU	1564 (1081-2005)	1339 (818-2194)	1431 (1099-1912)	0.71	0.84	260
Agatston score of aortic valve > 2000 AU in men, and > 1200 AU in women	19/50 (38%)	8/19 (42%)	19/43 (44%)	0.55	0.88	260
**Treatment strategy**						
Initial SAVR or TAVR strategy [a]	63 (41%)	20 (28%)	44 (30%)	0.034	0.76	0
Initial SAVR	21 (14%)	6 (8%)	16 (11%)			
Initial TAVR	42 (28%)	14 (19%)	28 (19%)			
Conservative strategy	89 (59%)	52 (72%)	104 (70%)	0.034	0.76	0
Watchful waiting	48 (32%)	37 (51%)	69 (47%)			
Patient rejection	17 (11%)	6 (8%)	12 (8%)			
Contraindication for both SAVR and TAVR	24 (16%)	9 (13%)	23 (16%)			

Categorical variables were presented as numbers and percentages. Continuous variables were expressed as the mean and SD or median and interquartile range (IQR).

[a] Risk-adjusting variables selected for the Cox proportional hazard model.

[b] Anemia was defined by the World Health Organization criteria (hemoglobin <12.0 g/dl in women and <13.0 g/dl in men).

[c] Clinical frailty scale scores were determined according to the Canadian Study of Health and Aging grading criteria.

All abbreviations were the same as in Table 1.

Table S2. Baseline clinical and echocardiographic characteristics according to the baseline left ventricular ejection fraction: HG AS.

HG AS	LVEF <50% N=207	LVEF 50-59% N=341	LVEF ≥60% (reference) N=1438	LVEF <50% versus LVEF ≥60%	LVEF 50-59% versus LVEF ≥60%	Missing value
**Clinical characteristics**						
Age, years [a]	81.6±8.1	81.5±8.4	81.0±8.5	0.37	0.33	0
Men [a]	106 (51%)	177 (52%)	503 (35%)	<0.001	<0.001	0
BMI, kg/m^2 [a]	22.2±3.6	22.6±3.5	22.9±3.7	0.01	0.20	7
BSA, m^2	1.55±0.20	1.55±0.19	1.53±0.19	0.20	0.03	7
Hypertension [a]	150 (72%)	285 (84%)	1176 (82%)	0.002	0.44	0
Current smoking [a]	9 (4%)	16 (5%)	57 (4%)	0.79	0.54	0
Diabetes	59 (29%)	102 (30%)	363 (25%)	0.32	0.08	0
On insulin therapy [a]	8 (4%)	16 (5%)	55 (4%)	0.98	0.46	0
Prior myocardial infarction [a]	26 (13%)	20 (6%)	34 (2%)	<0.001	<0.001	0
Coronary artery disease [a]	88 (43%)	127 (37%)	419 (29%)	<0.001	0.004	0
Prior symptomatic stroke [a]	34 (16%)	52 (15%)	165 (11%)	0.04	0.06	0
Atrial fibrillation or flutter [a]	49 (24%)	85 (25%)	229 (16%)	0.005	<0.001	0
Aortic/peripheral vascular disease [a]	20 (10%)	21 (6%)	71 (5%)	0.005	0.36	0
Creatinine level >2 mg/dl and non-dialysis [a]	13 (6%)	20 (6%)	24 (2%)	<0.001	<0.001	0
Dialysis [a]	13 (6%)	32 (9%)	85 (6%)	0.83	0.02	0
Anemia [a, b]	118 (57%)	196 (57%)	777 (54%)	0.42	0.25	0
Liver cirrhosis (Child-Pugh B/C) [a]	2 (1.0%)	3 (0.9%)	13 (0.9%)	0.93	0.97	0
Malignancy currently under treatment[a]	13 (6%)	19 (6%)	89 (6%)	0.96	0.67	0
Chronic lung disease (moderate or severe) [a]	11 (5%)	22 (6%)	84 (6%)	0.76	0.67	0

STS score (PROM), %	4.6 (2.9-7.0)	4.4 (2.7-6.2)	3.8 (2.5-5.7)	<0.001	0.007	0
Symptoms possibly related to AS	191 (92%)	247 (72%)	910 (63%)	<0.001	0.001	0
HF hospitalization at the index echocardiography [a]	62 (30%)	54 (16%)	81 (6%)	<0.001	<0.001	0
Clinical frailty scale [a, c] 1-3	91 (44%)	176 (52%)	800 (56%)	<0.001	<0.001	0
4-6	95 (46%)	131 (38%)	572 (40%)			
7-9	21 (10%)	34 (10%)	66 (5%)			
	N=182	N=283	N=1178			343
BNP, pg/ml	719 (393-1496)	322 (132-630)	125 (57-293)	<0.001	<0.001	
	N=30	N=37	N=164			1755
NT-proBNP, pg/ml	8484 (4006-12323)	2407 (618-5430)	779 (276-2009)	<0.001	<0.001	
**Etiology of aortic stenosis**						
Degenerative	188 (91%)	302 (89%)	1297 (90%)	0.50	0.009	0
Congenital (unicuspid, bicuspid, or quadricuspid)	19 (9%)	31 (9%)	125 (9%)			
Rheumatic	0	5 (1%)	15 (1%)			
Infective endocarditis	0	3 (1%)	0			
Other	0	0	1 (0.1%)			
**Echocardiographic variables**						
Vmax, m/s	4.7±0.6	4.6±0.6	4.7±0.6	0.77	0.27	0
Vmax >5 m/s	51 (25%)	74 (22%)	344 (24%)	0.82	0.38	0
Mean aortic PG, mm Hg	53±15	51±14	51±15	0.23	0.78	4
AVA (equation of continuity), cm^2	0.56±0.20	0.66±0.20	0.73±0.20	<0.001	<0.001	7
Indexed AVA, cm^2/m^2	0.37±0.12	0.43±0.12	0.48±0.13	<0.001	<0.001	14
LV end-diastolic diameter, mm	50.8±7.1	46.9±6.0	43.2±5.3	<0.001	<0.001	1
LV end-systolic diameter, mm	39.8±7.4	32.4±4.9	26.8±4.2	<0.001	<0.001	20
LVEF, %	41±7	56±3	67±5	<0.001	<0.001	0
High LV mass index (men >115 g/m^2, and women >95g/m^2)	163/205 (80%)	222/339 (65%)	757/1412 (54%)	<0.001	<0.001	30
Relative wall thickness >0.42	92/207 (44%)	210/339 (62%)	1061/1417 (75%)	<0.001	<0.001	23
LV remodeling pattern						
Concentric hypertrophy	72/205 (35%)	148/339 (44%)	612/1412 (43%)	<0.001	<0.001	30

Concentric remodeling	19/205 (9%)	62/339 (18%)	444/1412 (31%)			
Eccentric hypertrophy	91/205 (44%)	74/339 (22%)	145/1412 (10%)			
Normal	23/205 (11%)	55/339 (16%)	211/1412 (15%)			
LVOT, mm	21.0±2.1	20.9±1.9	20.2±2.0	<0.001	<0.001	9
SV index, ml/m^2	40±12	46±11	51±12	<0.001	<0.001	14
Septal E/e'>15	144/171 (84%)	226/296 (76%)	887/1264 (70%)	<0.001	0.03	255
Cardiac damage						
Stage 0	0	19 (6%)	128 (9%)	<0.001	<0.001	0
Stage 1	37 (18%)	56 (16%)	391 (27%)			
Stage 2	145 (70%)	234 (69%)	806 (56%)			
Stage 3-4	25 (12%)	32 (9%)	113 (8%)			
Any combined valvular disease (moderate or severe) [a]	67 (32%)	109 (32%)	320 (22%)	0.001	<0.001	0
Moderate or severe AR	30 (14%)	59 (17%)	153 (11%)	0.10	<0.001	0
Moderate or severe MS	6 (3%)	6 (2%)	51 (4%)	0.63	0.09	0
Moderate or severe MR	31 (15%)	50 (15%)	114 (8%)	<0.001	<0.001	0
Moderate or severe TR	21 (10%)	25 (7%)	104 (7%)	0.14	0.95	0
TR pressure gradient ≥40 mm Hg [a]	42 (20%)	51 (15%)	107 (7%)	<0.001	<0.001	0
**CT variables**						
Agatston score of aortic valve, AU	3087 (2341-4168)	2361 (1724-3256)	2063 (1437-2774)	<0.001	0.003	1200
Agatston score of aortic valve > 2000 AU in men, and > 1200 AU in women	73/80 (91%)	114/134 (85%)	464/572 (81%)	0.03	0.28	1200
**Treatment strategy**						
Initial SAVR or TAVR strategy [a]	161 (78%)	229 (67%)	987 (69%)	0.007	0.60	0
Initial SAVR	57 (28%)	79 (23%)	335 (23%)			
Initial TAVR	104 (50%)	150 (44%)	652 (45%)			
Conservative strategy	46 (22%)	112 (33%)	451 (31%)	0.007	0.60	0
Watchful waiting	11 (5%)	54 (16%)	287 (20%)			
Patient rejection	17 (8%)	27 (8%)	102 (7%)			
Contraindication for both SAVR and TAVR	18 (9%)	31 (9%)	62 (4%)			

HG= high gradient.

Other abbreviations were the same as in Supplementary Table 1.

Figure S1. Kaplan-Meier curves for SAVR or TAVR according to the baseline left ventricular ejection fraction.

(A) Low gradient AS with normal flow, (B) Low gradient with low flow, and (C) High gradient AS. SAVR=surgical aortic valve replacement; transcatheter aortic valve replacement.

(A) LG AS with Normal Flow

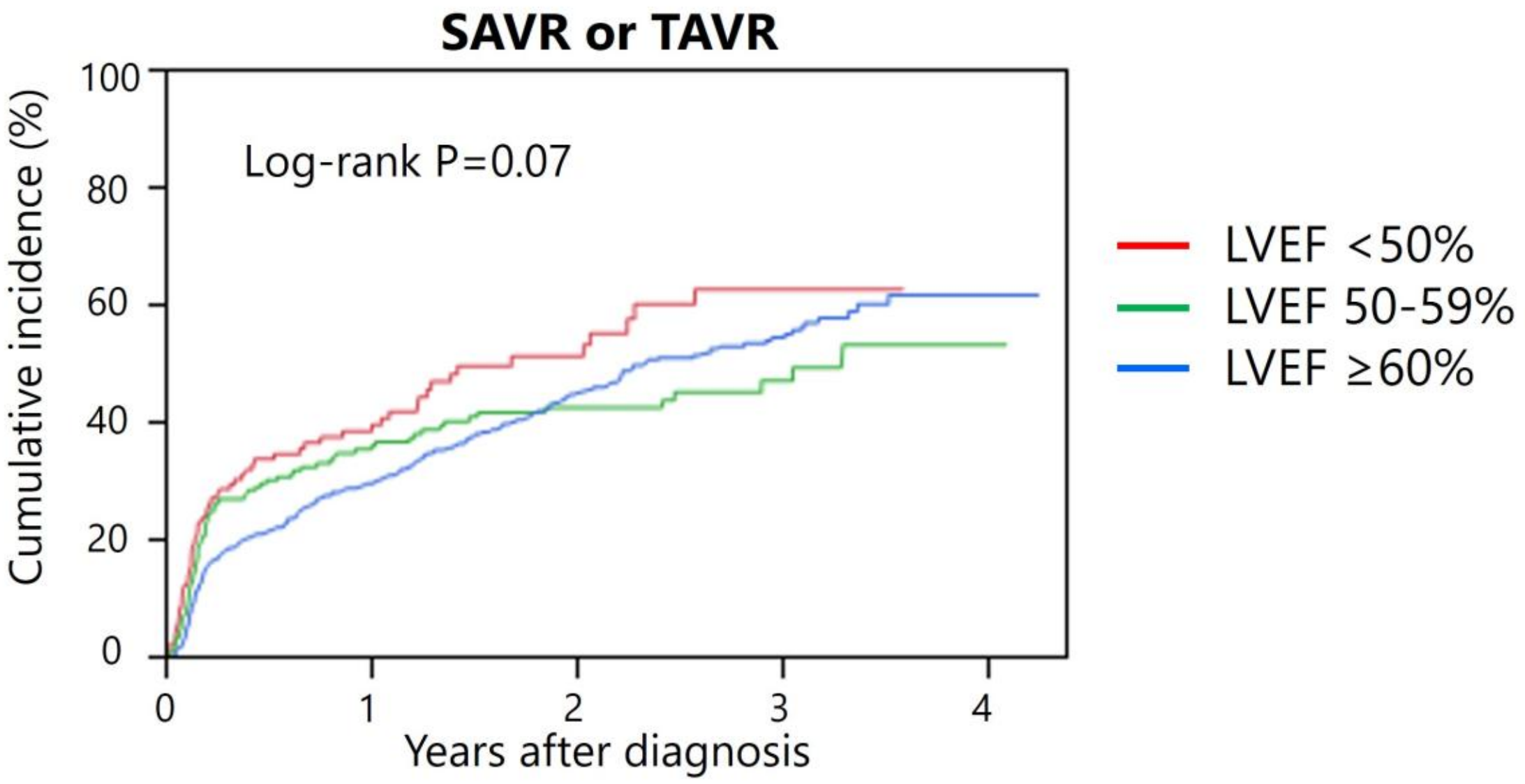

Interval	0 day	1 year	2 years	3 years
LVEF <50%				
N of patients with SAVR or TAVR		50	59	64
N of patients at risk	133	54	25	8
Cumulative incidence		40.2%	51.9%	63.3%
LVEF 50-59%				
N of patients with SAVR or TAVR		71	79	82
N of patients at risk	201	101	56	23
Cumulative incidence		37.3%	43.1%	47.7%
LVEF ≥60%				
N of patients with SAVR or TAVR		197	271	300
N of patients at risk	671	399	216	74
Cumulative incidence		30.8%	45.8%	55.7%

(B) LG AS with Low Flow

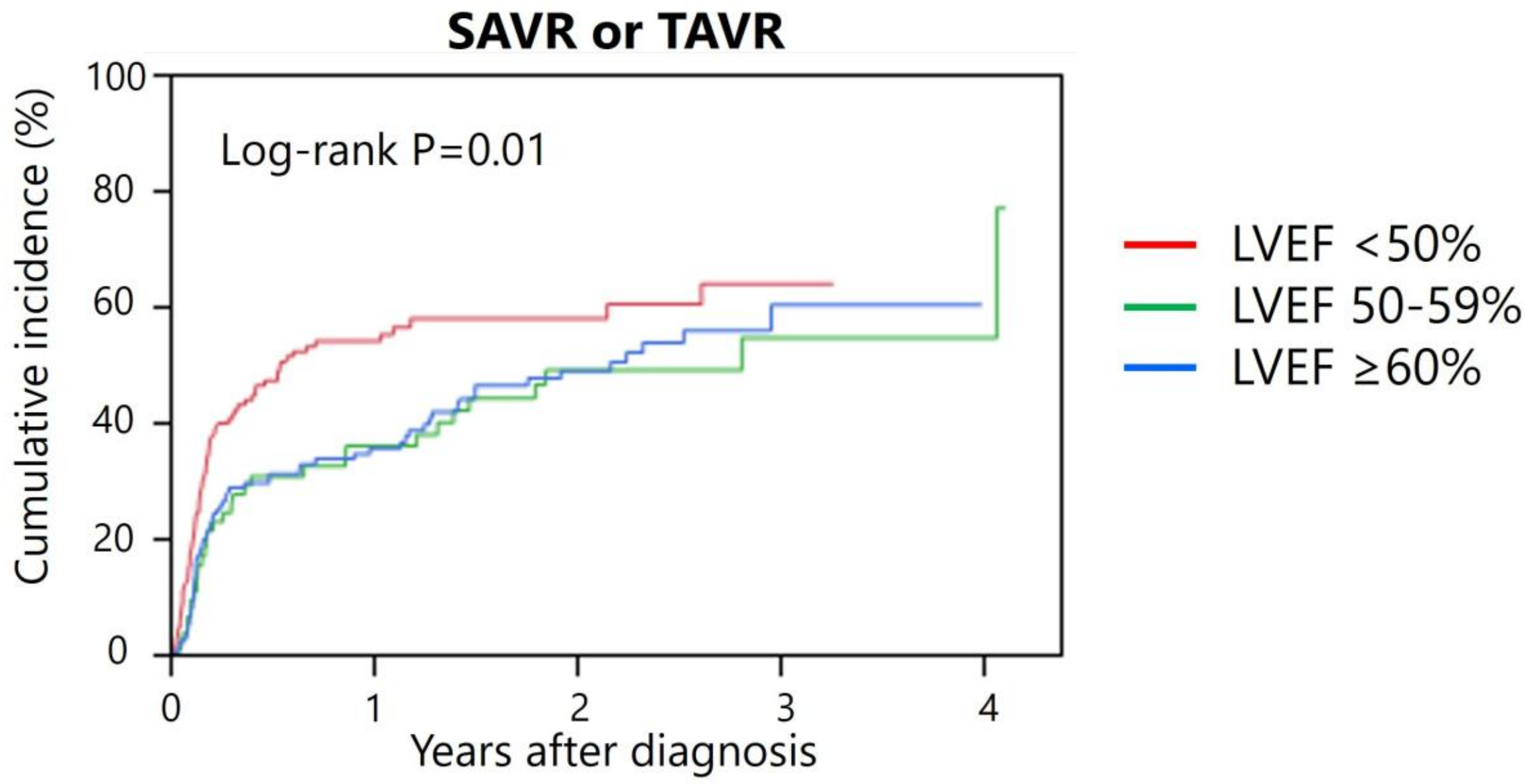

Interval	0 day	1 year	2 years	3 years
**LVEF <50%**				
N of patients with SAVR or TAVR		73	76	78
N of patients at risk	152	38	17	5
Cumulative incidence		54.8%	58.7%	64.7%
**LVEF 50-59%**				
N of patients with SAVR or TAVR		24	30	31
N of patients at risk	72	35	18	5
Cumulative incidence		36.7%	49.8%	55.3%
**LVEF ≥60%**				
N of patients with SAVR or TAVR		50	62	67
N of patients at risk	148	66	37	8
Cumulative incidence		36.3%	49.7%	61.0%

(C) HG AS

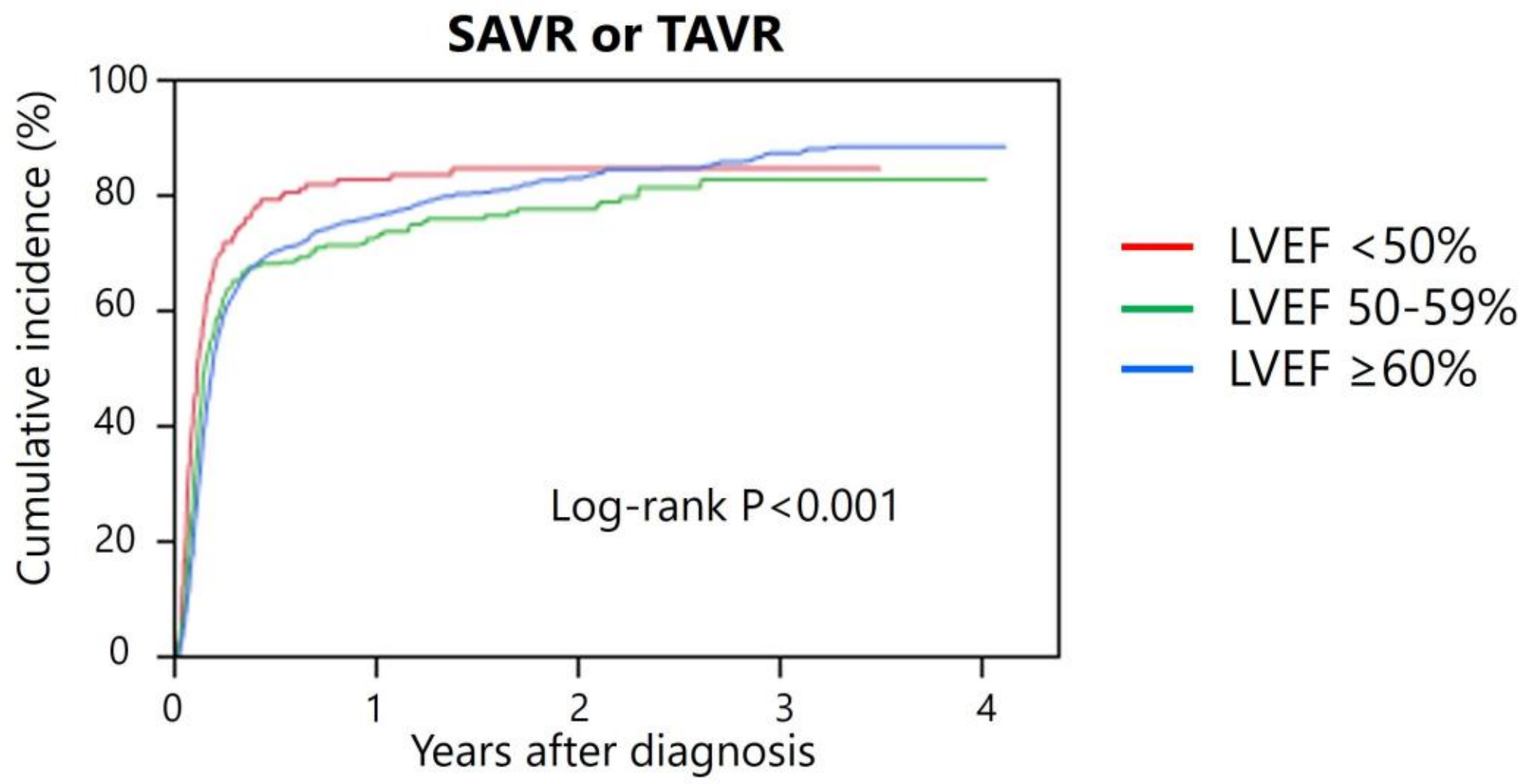

Interval	0 day	1 year	2 years	3 years
LVEF <50%				
N of patients with SAVR or TAVR		165	167	167
N of patients at risk	207	19	9	3
Cumulative incidence		83.4%	85.3%	85.3%
LVEF 50-59%				
N of patients with SAVR or TAVR		241	250	256
N of patients at risk	341	66	35	9
Cumulative incidence		74.0%	78.3%	83.4%
LVEF ≥60%				
N of patients with SAVR or TAVR		1080	1143	1165
N of patients at risk	1438	259	114	40
Cumulative incidence		77.2%	83.7%	88.0%

Figure S2. Kaplan-Meier curves for a composite of death or heart failure hospitalization according to the baseline left ventricular ejection fraction in patients with Low gradient AS with normal flow in the initial AVR stratum (A) and in the conservative stratum (B).

LG AS with Normal Flow

(A) Initial AVR stratum

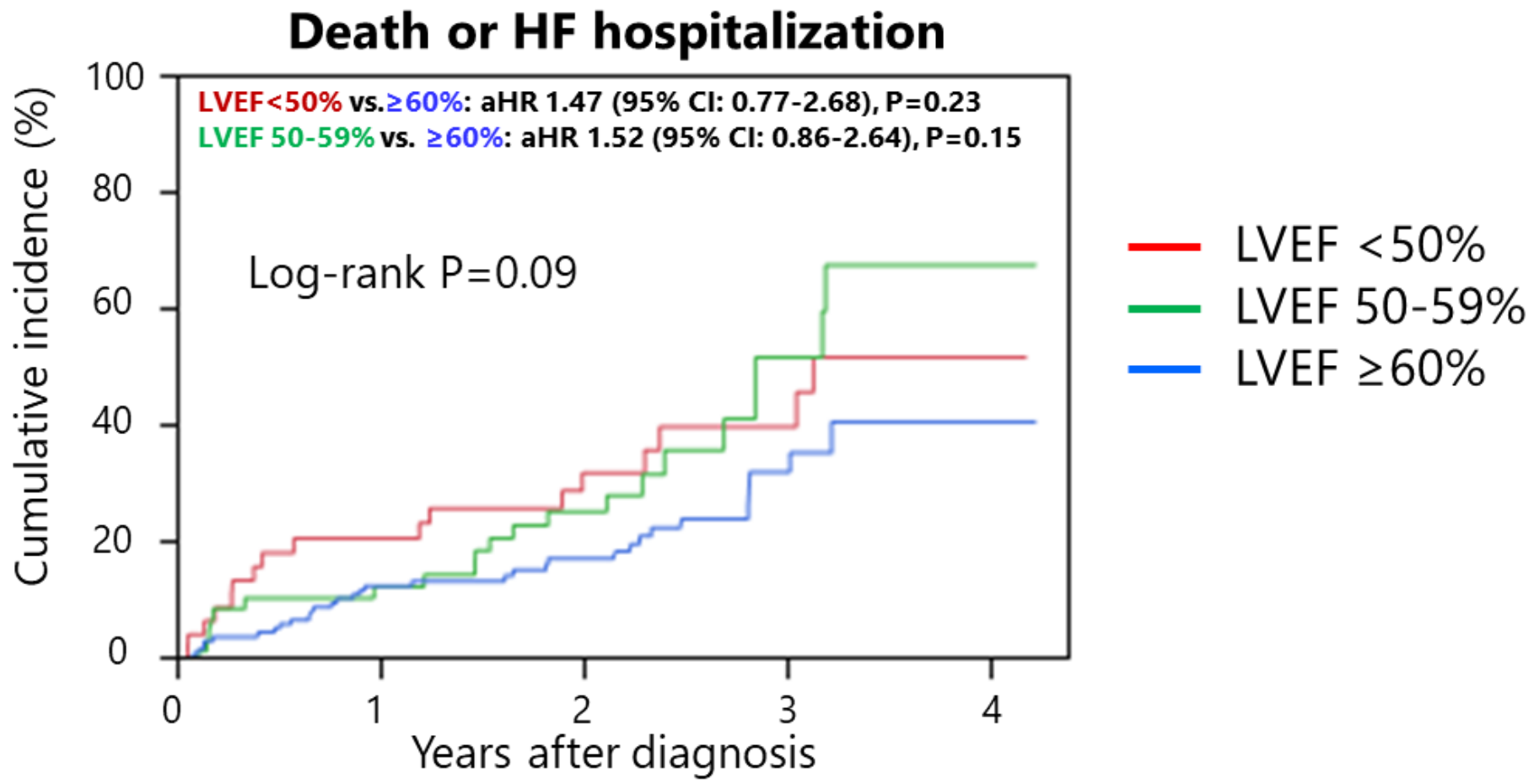

Interval	0 day	1 year	2 years	3 years
LVEF <50%				
N of patients with at least 1 event		9	13	15
N of patients at risk	43	32	21	10
Cumulative incidence		21.1%	32.4%	40.4%
LVEF 50-59%				
N of patients with at least 1 event		7	13	19
N of patients at risk	55	45	29	6
Cumulative incidence		12.9%	25.8%	52.3%
LVEF ≥60%				
N of patients with at least 1 event		18	23	32
N of patients at risk	140	117	75	20
Cumulative incidence		13.1%	17.7%	35.8%

(B) Conservative stratum

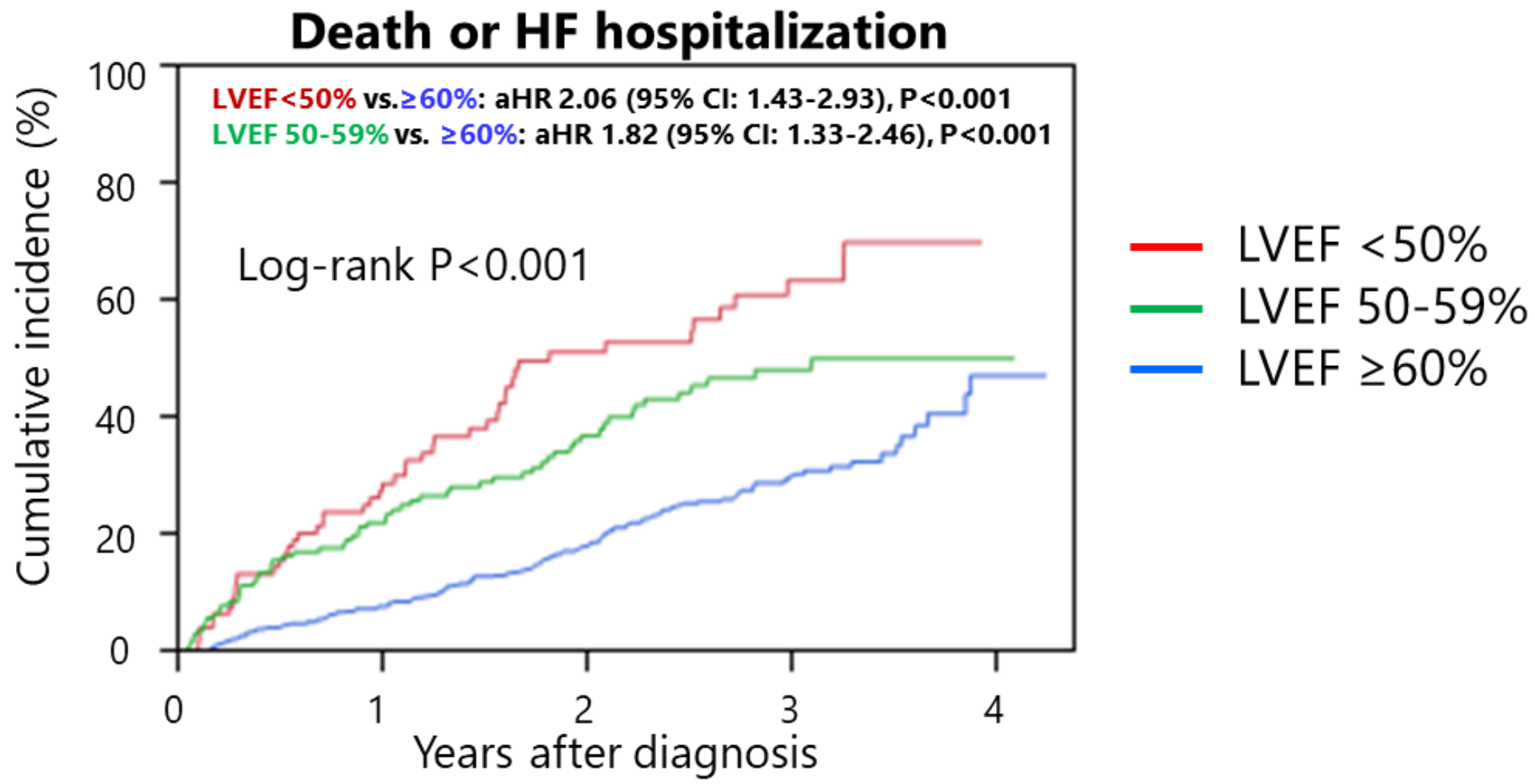

Interval	0 day	1 year	2 years	3 years
LVEF <50%				
N of patients with at least 1 event		25	41	47
N of patients at risk	90	56	30	14
Cumulative incidence		29.2%	51.6%	63.8%
LVEF 50-59%				
N of patients with at least 1 event		33	50	60
N of patients at risk	146	106	63	29
Cumulative incidence		23.2%	37.3%	48.6%
LVEF ≥60%				
N of patients with at least 1 event		41	87	122
N of patients at risk	531	437	305	133
Cumulative incidence		8.1%	18.8%	30.8%

Figure S3. Kaplan-Meier curves for a composite of death or heart failure hospitalization according to the baseline left ventricular ejection fraction in patients with Low gradient AS with low flow in the initial AVR stratum (A) and in the conservative stratum (B).

LG AS with Low Flow

(A) Initial AVR stratum

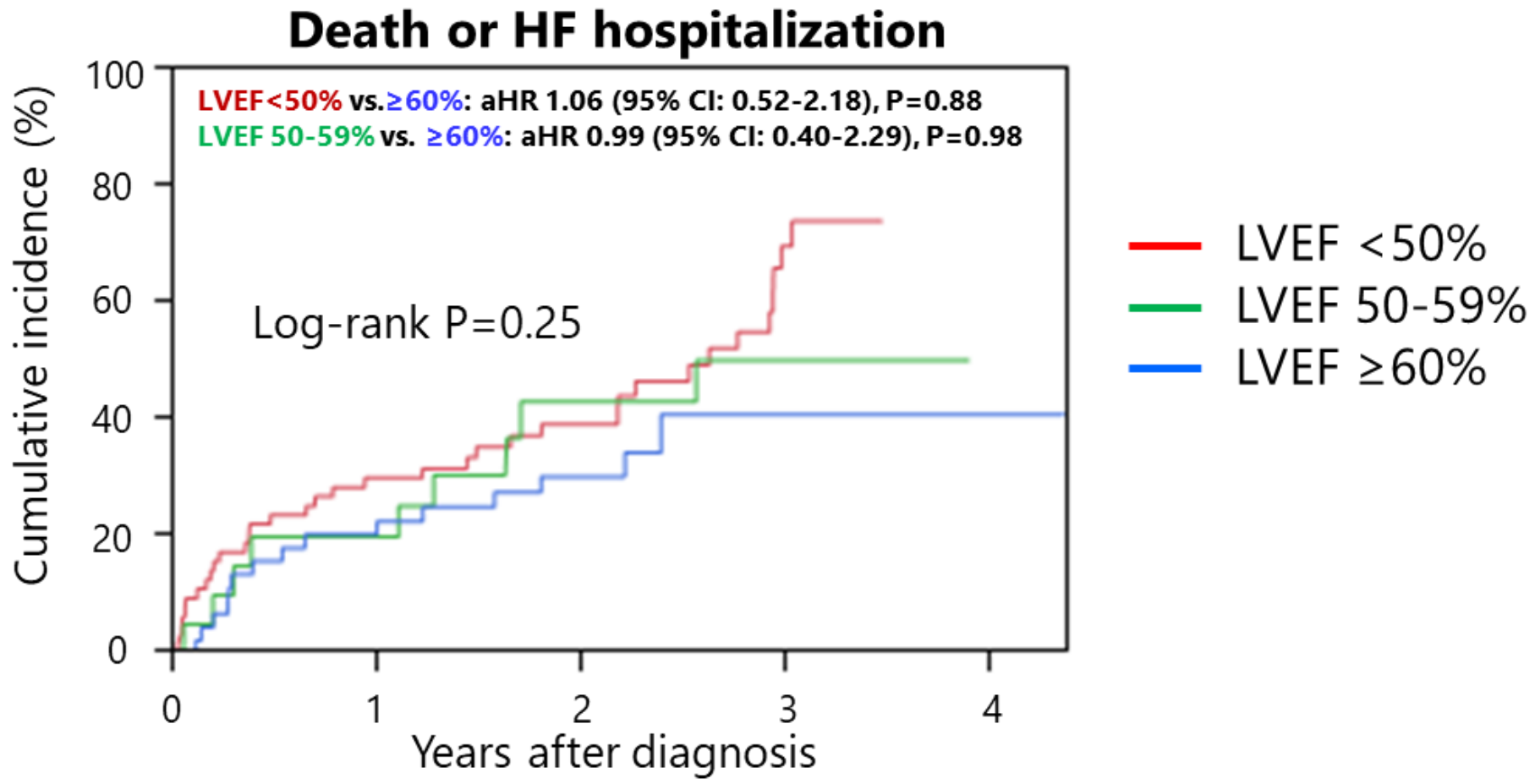

Interval	0 day	1 year	2 years	3 years
LVEF <50%				
N of patients with at least 1 event		19	24	34
N of patients at risk	63	44	28	7
Cumulative incidence		30.2%	39.4%	69.9%
LVEF 50-59%				
N of patients with at least 1 event		4	8	9
N of patients at risk	20	15	9	6
Cumulative incidence		20.0%	43.3%	50.4%
LVEF ≥60%				
N of patients with at least 1 event		10	13	15
N of patients at risk	44	34	24	4
Cumulative incidence		22.7%	30.4%	41.0%

(B) Conservative stratum

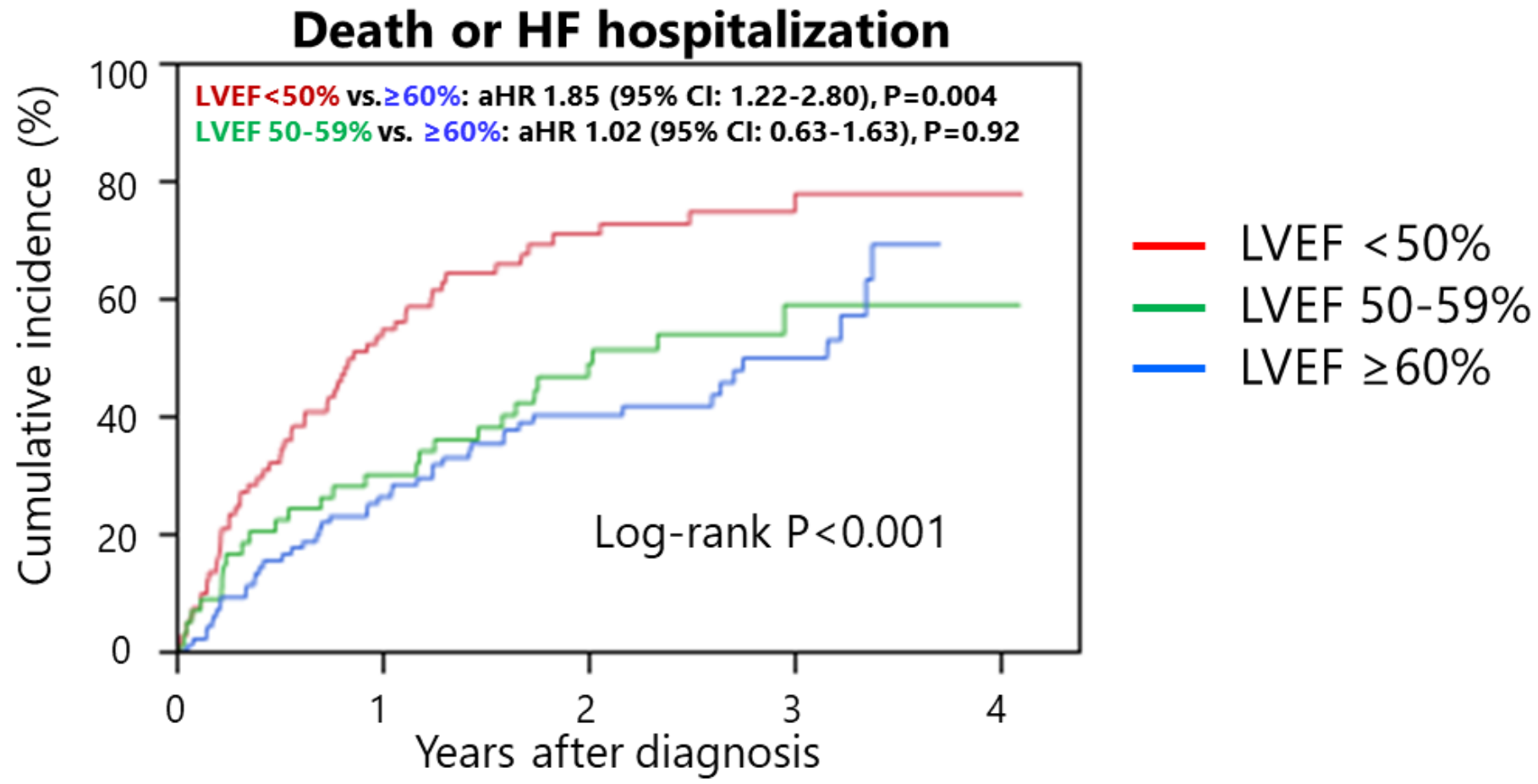

Interval	0 day	1 year	2 years	3 years
LVEF <50%				
N of patients with at least 1 event		45	56	59
N of patients at risk	89	35	16	6
Cumulative incidence		55.5%	71.7%	78.6%
LVEF 50-59%				
N of patients with at least 1 event		16	26	28
N of patients at risk	52	36	22	7
Cumulative incidence		30.8%	52.0%	59.7%
LVEF ≥60%				
N of patients with at least 1 event		26	38	43
N of patients at risk	104	68	42	18
Cumulative incidence		27.0%	41.0%	50.6%

Figure S4. Kaplan-Meier curves for a composite of death or heart failure hospitalization according to the baseline left ventricular ejection fraction in patients with High gradient AS in the initial AVR stratum (A) and in the conservative stratum (B).

HG AS

(A) Initial AVR stratum

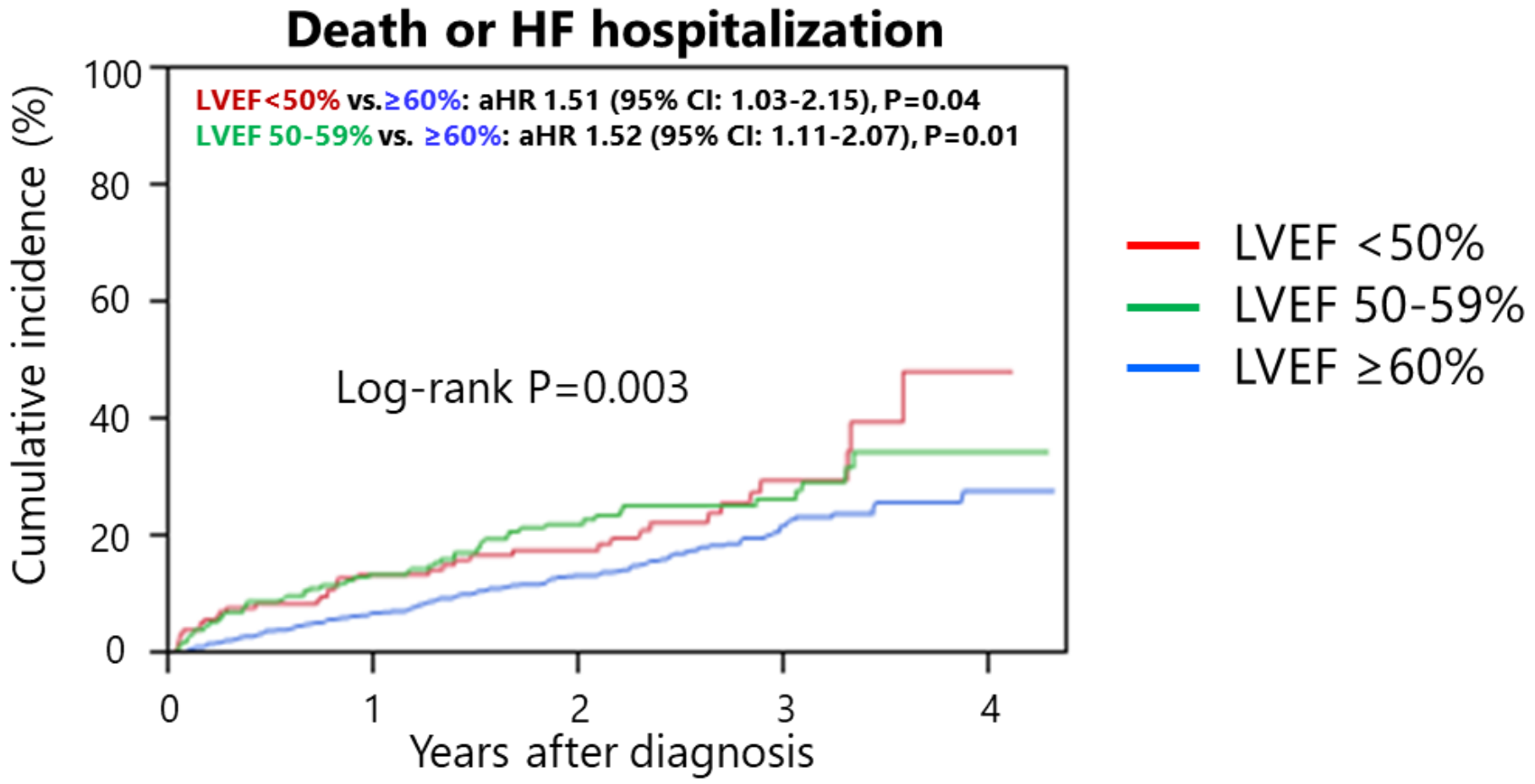

Interval	0 day	1 year	2 years	3 years
LVEF <50%				
N of patients with at least 1 event		22	27	35
N of patients at risk	161	132	85	29
Cumulative incidence		13.9%	18.1%	29.9%
LVEF 50-59%				
N of patients with at least 1 event		31	46	51
N of patients at risk	229	179	107	54
Cumulative incidence		13.8%	22.5%	26.7%
LVEF ≥60%				
N of patients with at least 1 event		70	116	148
N of patients at risk	987	859	516	209
Cumulative incidence		7.3%	13.7%	22.2%

(B) Conservative stratum

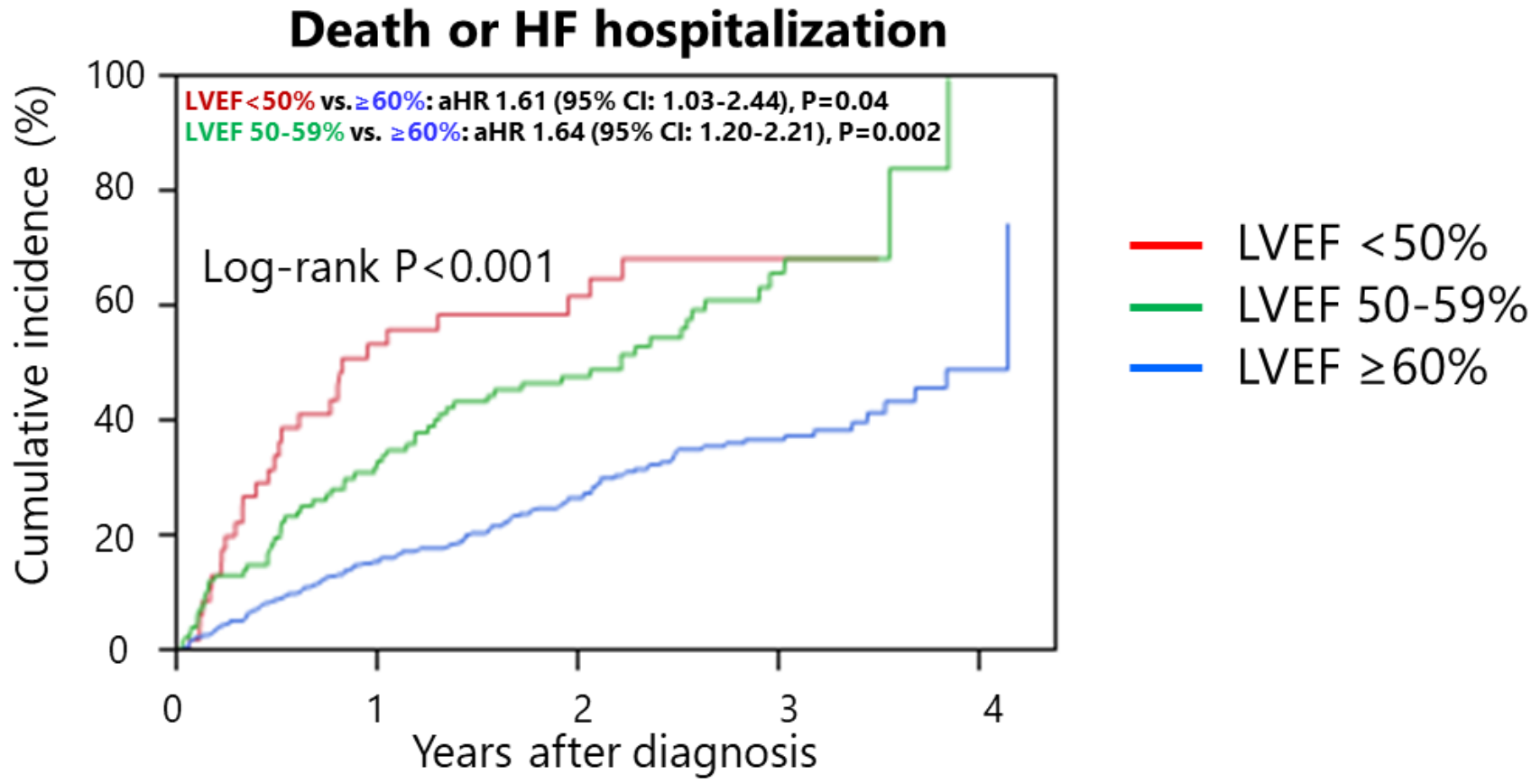

Interval	0 day	1 year	2 years	3 years
LVEF <50%				
N of patients with at least 1 event		23	26	28
N of patients at risk	46	18	12	4
Cumulative incidence		53.8%	62.1%	68.7%
LVEF 50-59%				
N of patients with at least 1 event		36	50	61
N of patients at risk	112	67	46	14
Cumulative incidence		33.4%	48.2%	66.1%
LVEF ≥60%				
N of patients with at least 1 event		71	105	129
N of patients at risk	451	326	198	84
Cumulative incidence		16.7%	27.1%	37.1%

Figure S5. Kaplan-Meier curves for death or HF hospitalization according to the baseline left ventricular ejection fraction categories in patients without prior myocardial infarction.

(A) Low gradient AS with normal flow, (B) Low gradient with low flow, and (C) High gradient AS. Cumulative incidence was estimated at 3 years, while the number of patients with event was counted throughout the entire follow-up period.

CI=confidence interval; HF=heart failure; HR=hazard ratio.

(A) LG AS with Normal Flow

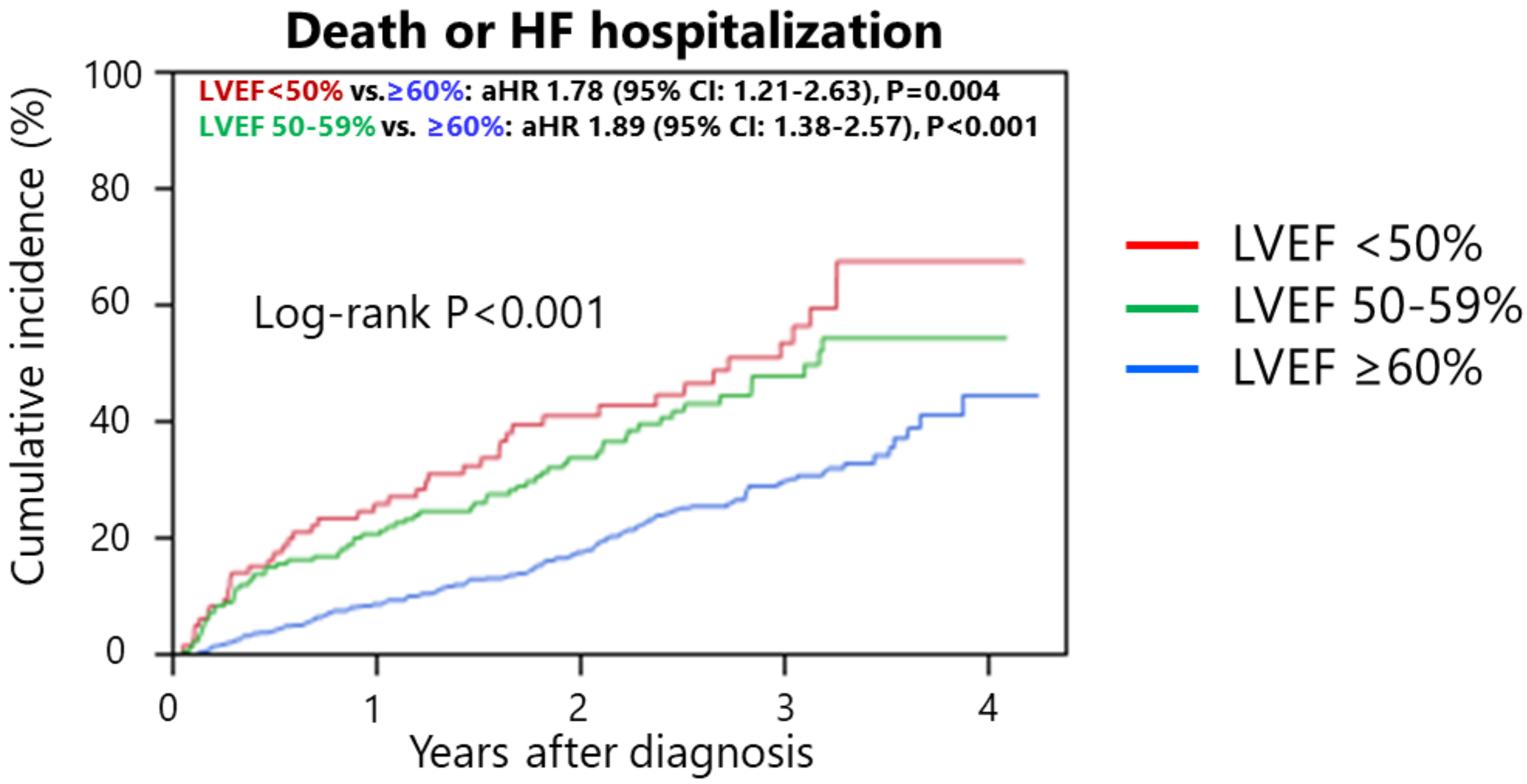

Interval	0 day	1 year	2 years	3 years
**LVEF <50%**				
N of patients with at least 1 event		23	34	40
N of patients at risk	91	59	35	17
Cumulative incidence		26.4%	41.6%	54.1%
**LVEF 50-59%**				
N of patients with at least 1 event		36	53	65
N of patients at risk	169	123	75	27
Cumulative incidence		21.9%	34.4%	48.3%
**LVEF ≥60%**				
N of patients with at least 1 event		55	101	142
N of patients at risk	627	516	357	146
Cumulative incidence		9.2%	18.3%	30.8%

(B) LG AS with Low Flow

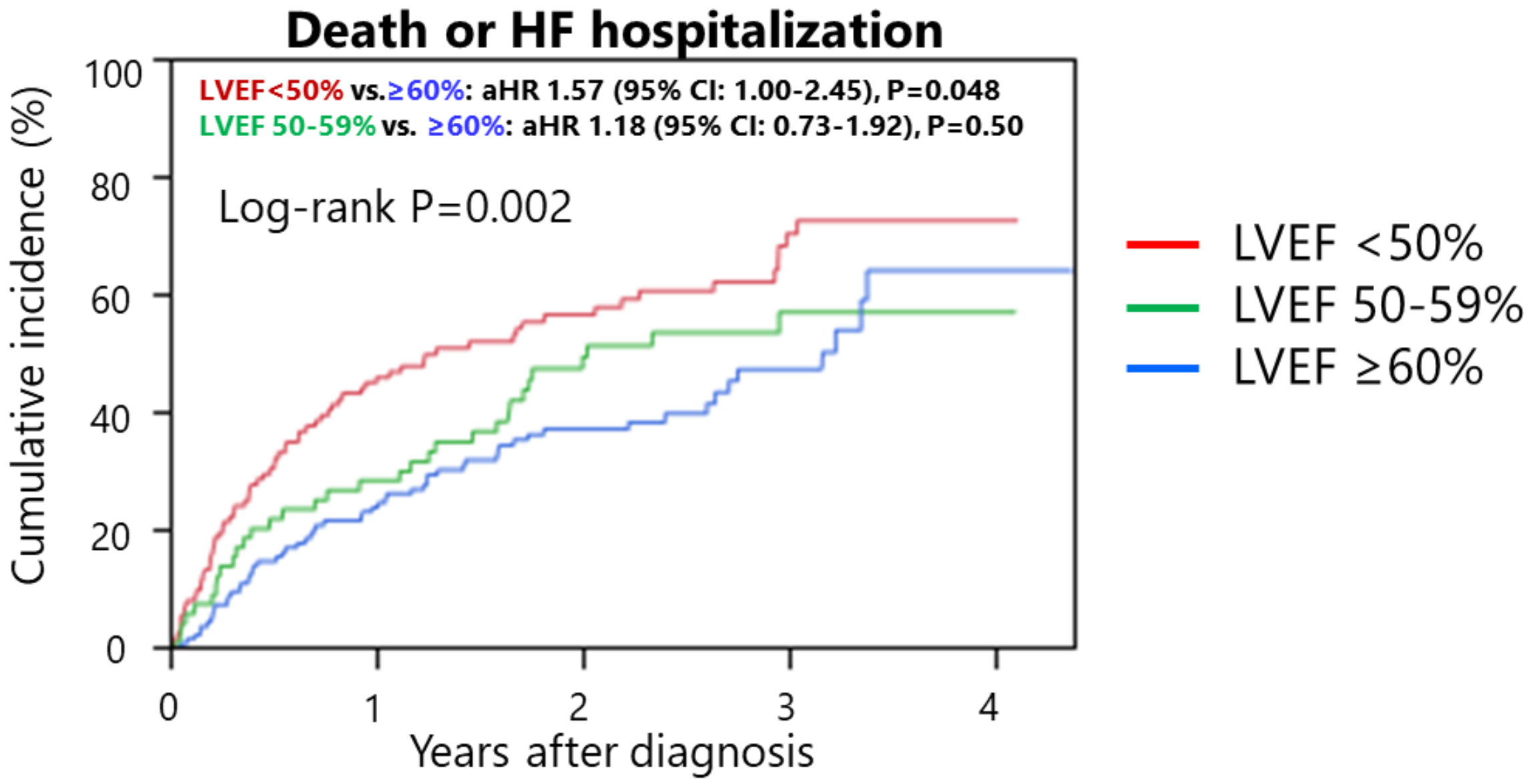

Interval	0 day	1 year	2 years	3 years
LVEF <50%				
N of patients with at least 1 event		52	62	70
N of patients at risk	119	58	34	13
Cumulative incidence		46.7%	57.3%	71.1%
LVEF 50-59%				
N of patients with at least 1 event		18	31	33
N of patients at risk	62	44	26	11
Cumulative incidence		29.0%	52.0%	57.7%
LVEF ≥60%				
N of patients with at least 1 event		34	49	55
N of patients at risk	142	98	63	21
Cumulative incidence		25.3%	37.8%	48.1%

(C) HG AS

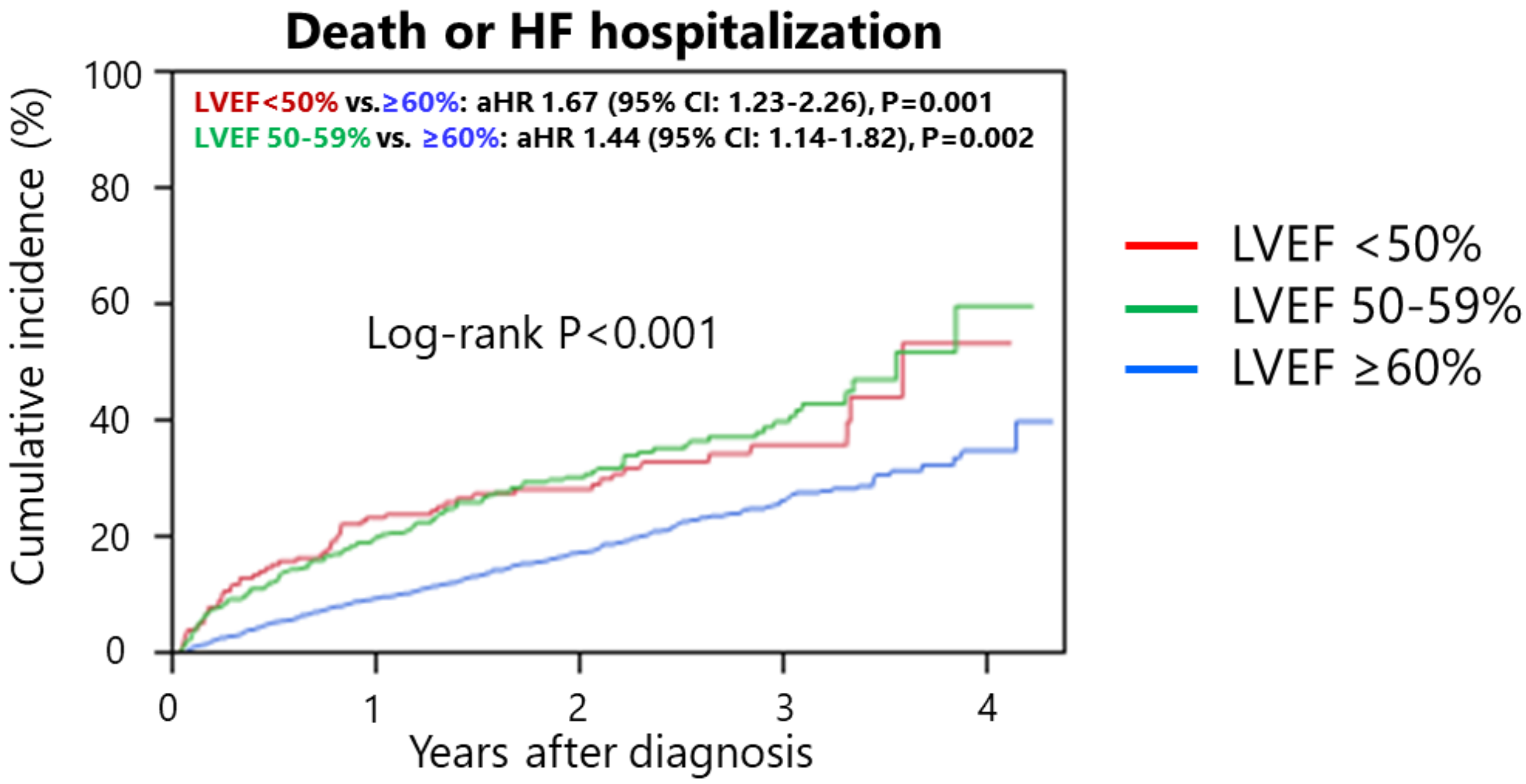

Interval	0 day	1 year	2 years	3 years
**LVEF <50%**				
N of patients with at least 1 event		42	49	56
N of patients at risk	181	129	85	30
Cumulative incidence		23.8%	28.7%	36.3%
**LVEF 50-59%**				
N of patients with at least 1 event		64	90	105
N of patients at risk	321	230	143	63
Cumulative incidence		20.5%	30.8%	40.3%
**LVEF ≥60%**				
N of patients with at least 1 event		138	215	270
N of patients at risk	1404	1155	698	289
Cumulative incidence		10.2%	17.8%	26.8%

Figure S6. Kaplan-Meier curves for death or HF hospitalization according to the baseline left ventricular ejection fraction categories in patients whose LVEFs were measured using the biplane Simpson method of disks.

(A) Low gradient AS with normal flow, (B) Low gradient with low flow, and (C) High gradient AS. Cumulative incidence was estimated at 3 years, while the number of patients with event was counted throughout the entire follow-up period.

(A) LG AS with Normal Flow

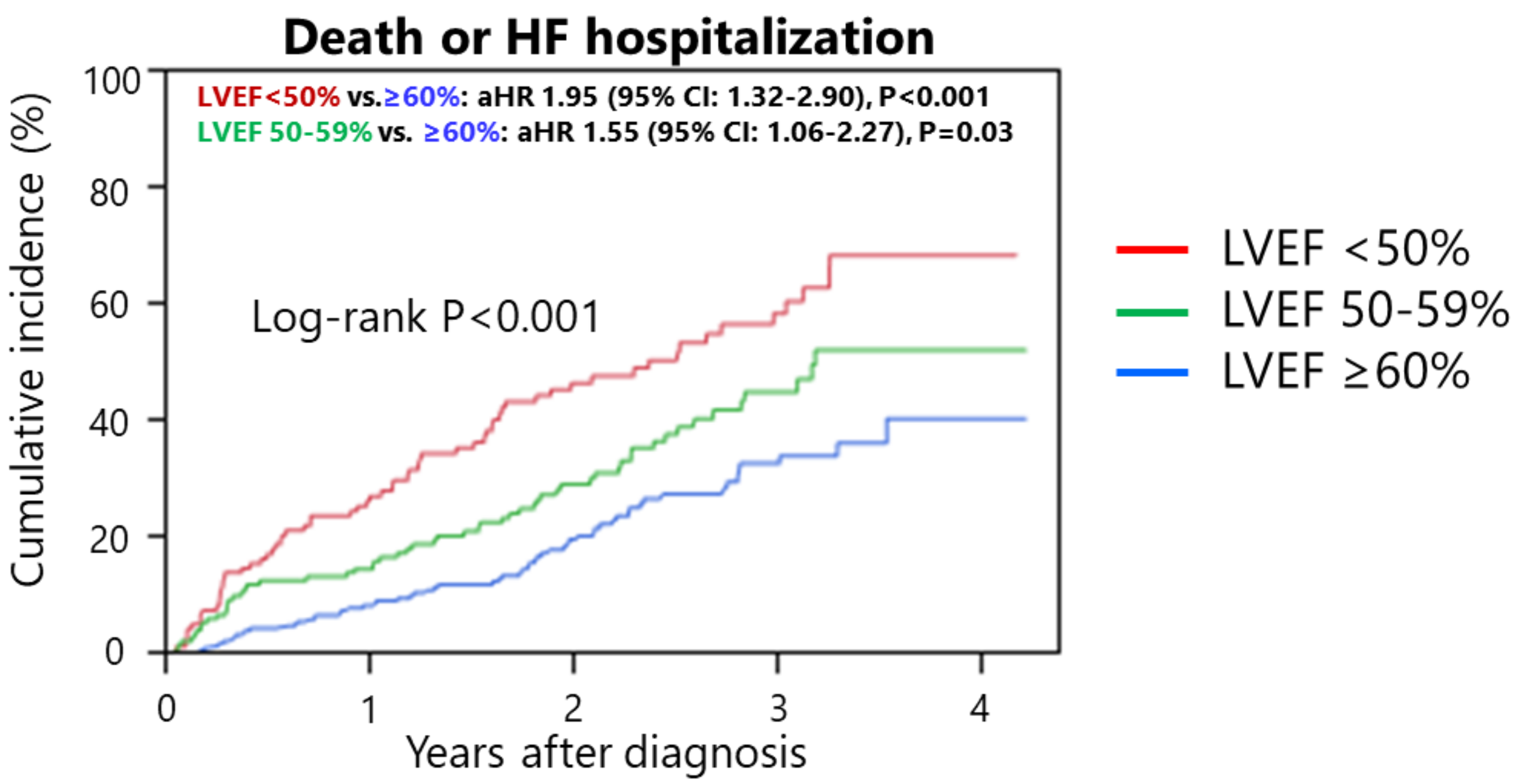

Interval	0 day	1 year	2 years	3 years
LVEF <50%				
N of patients with at least 1 event		34	54	62
N of patients at risk	128	84	47	21
Cumulative incidence		27.5%	46.9%	58.9%
LVEF 50-59%				
N of patients with at least 1 event		24	42	55
N of patients at risk	157	124	76	26
Cumulative incidence		15.7%	29.5%	45.4%
LVEF ≥60%				
N of patients with at least 1 event		23	45	62
N of patients at risk	284	220	128	52
Cumulative incidence		8.7%	20.1%	34.3%

(B) LG AS with Low Flow

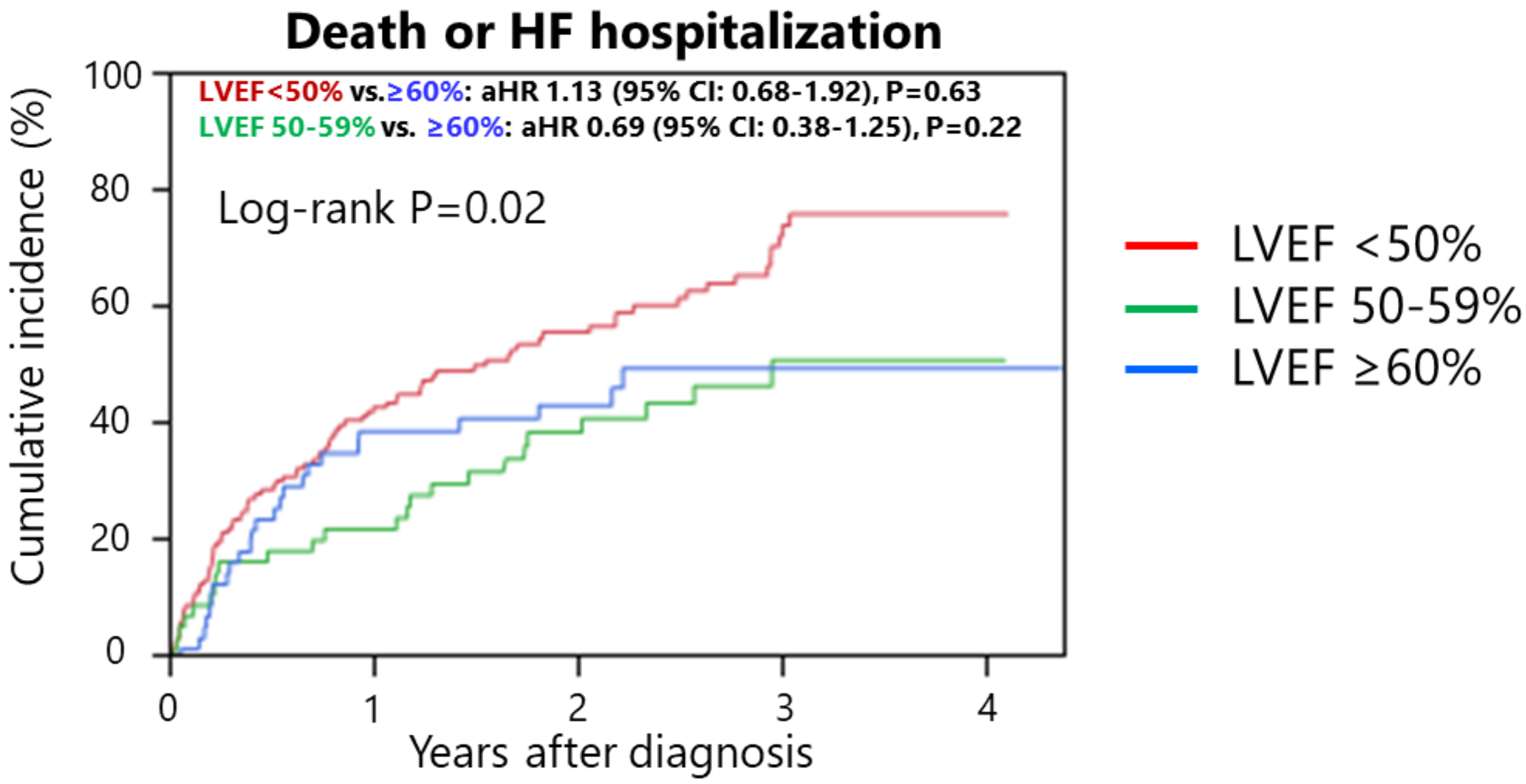

Interval	0 day	1 year	2 years	3 years
**LVEF <50%**				
N of patients with at least 1 event		59	74	87
N of patients at risk	143	76	42	13
Cumulative incidence		43.2%	56.1%	74.5%
**LVEF 50-59%**				
N of patients with at least 1 event		12	21	24
N of patients at risk	54	41	26	10
Cumulative incidence		22.3%	41.3%	51.3%
**LVEF ≥60%**				
N of patients with at least 1 event		21	23	25
N of patients at risk	57	32	23	4
Cumulative incidence		39.2%	43.6%	49.9%

(C) HG AS

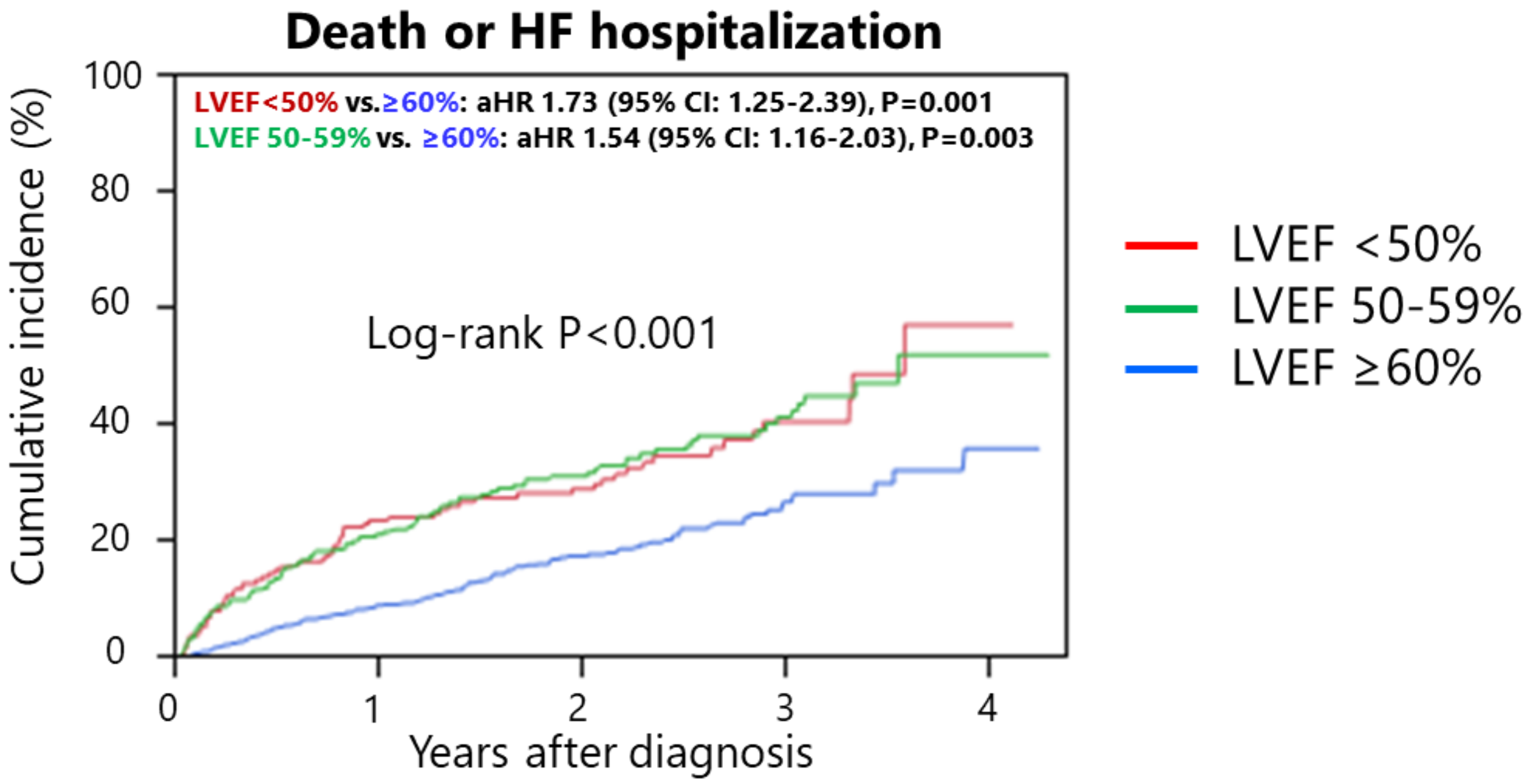

Interval	0 day	1 year	2 years	3 years
**LVEF <50%**				
N of patients with at least 1 event		45	53	63
N of patients at risk	193	137	88	28
Cumulative incidence		23.9%	29.4%	40.9%
**LVEF 50-59%**				
N of patients with at least 1 event		55	76	89
N of patients at risk	261	188	119	53
Cumulative incidence		21.6%	31.6%	41.7%
**LVEF ≥60%**				
N of patients with at least 1 event		70	108	129
N of patients at risk	774	616	300	104
Cumulative incidence		9.5%	17.8%	27.1%
